# Triplet-based species tree estimation: Sensitivity to gene tree rooting (or lack thereof)

**DOI:** 10.1371/journal.pcbi.1014782

**Published:** 2026-09-23

**Authors:** Tanjeem Azwad Zaman, Rabib Jahin Ibn Momin, Md Shamsuzzoha Bayzid

**Affiliations:** Department of Computer Science and Engineering, Bangladesh University of Engineering and Technology, Dhaka, Bangladesh; University of Auckland, NEW ZEALAND

## Abstract

Species tree estimation often involves phylogenomic methodologies that incorporate multiple genes sampled across the genome. However, gene tree heterogeneity (discordance), often attributed to Incomplete Lineage Sorting (ILS), presents a significant challenge to accurate species tree inference. Triplet and quartet-based species tree estimation methods have attracted considerable attention owing to their provable statistical consistency in the presence of ILS. Yet, the adoption of rooted triplet-based approaches in the systematics community has been restricted, largely due to their limitations in handling unrooted gene trees—an issue not faced by quartet-based methods. Since the root placement directly influences the distribution of induced triplets in a gene tree, the accuracy of triplet-based methods is dependent on gene tree rooting accuracy. While there is extensive research on approaches for rooting unrooted gene trees, the choice of rooting techniques and their implications for triplet-based species tree estimation on realistic model conditions are greatly understudied. In this study, we carry out an extensive empirical analysis of different gene tree rooting strategies to assess the effects of rooting on triplet-based species tree inference. Across simulated and empirical datasets, triplet-based estimation using STELAR is generally robust to gene tree rooting, with several algorithmic rooting strategies yielding performance comparable to, and in some model conditions exceeding, that of STELAR on outgroup-rooted gene trees and the widely used quartet-based method ASTRAL. Our results highlight different model conditions in which rooting choices positively influence triplet-based species tree inference, thereby providing new insights into their potential utility in phylogenomic analyses.

## Author summary

Reconstructing the evolutionary history of species from genomic data is complicated by the fact that different genes can tell different evolutionary stories – a problem known as gene tree discordance. To address this, researchers use methods that identify consistent patterns across many gene trees, comparing either sets of four (quartets) or three species (triplets). Triplet-based methods have seen less adoption partly because they require gene trees to be “rooted” – meaning the direction of evolution must be specified – while quartet-based methods do not. Rooting is typically done using an outgroup, but a reliable one isn’t always available. In this study, we conducted an extensive empirical evaluation of how different rooting strategies affect STELAR – a triplet-based species tree estimation method – across various simulated and biological datasets, spanning diverse model conditions and including varying levels of gene tree discordance, gene counts, and sequence lengths. We found that STELAR is generally robust to the choice of rooting method – algorithmic rooting approaches often performed comparably to, and sometimes better than, outgroup rooting or the popular quartet-based method ASTRAL. These findings suggest that the lack of a reliable outgroup need not be a barrier to using triplet-based methods and open new avenues for improving their performance in phylogenomic studies.

## 1 Introduction

Species tree estimation from multiple loci is increasingly prevalent. However, combining multi-locus data is difficult, especially in the presence of gene tree discordance, where different genes may have different evolutionary histories. A traditional approach for species tree estimation from multiple genes is called concatenation (also called ‘combined analysis’), where alignments of the genes are concatenated into a supermatrix, which is then used to estimate the species tree. Although it is a widely used technique, concatenation can be problematic as it is agnostic to the topological differences among the gene trees, can be statistically inconsistent [1], and can return incorrect trees with high confidence [2–5]. As a result, “summary methods”, which operate by computing gene trees from different loci and then combining the inferred gene trees into a species tree, are becoming increasingly popular [6].

Recent modeling and computational advances have produced summary methods that explicitly take gene tree discordance into account while estimating species trees from multi-locus data. While several biological processes, such as gene duplication and loss, incomplete lineage sorting (ILS), and horizontal gene transfer, can result in this gene tree conflict, ILS (modeled by the multi-species coalescent [7]) has been recognized as a major contributor to gene tree heterogeneity, particularly in closely related species that evolve with rapid speciation events (i.e., rapid radiations) [8]. Consequently, a substantial body of recent literature has focused on estimating species trees under the MSC framework to account for discordance caused by ILS, alongside parallel efforts that explicitly model gene duplication, loss, and transfer. Quartet and triplet-based summary methods [9–17] have gained significant attention as quartets (4-leaf unrooted trees) and triplets (3-leaf rooted trees) do not contain the “anomaly zone” [18–20], a condition where the most probable gene tree topology may differ from the species tree topology. ASTRAL [21–23], the most widely used summary method, is a quartet-based method that uses a dynamic programming (DP) approach to find a species tree that is consistent with the largest number of quartets induced by the set of gene trees. STELAR [17], on the other hand, is a triplet-based method which seeks a species tree by maximizing the number of consistent triplets with respect to the input gene trees. Both ASTRAL and STELAR are statistically consistent, have comparable accuracy, and are scalable to large datasets. However, while ASTRAL has been a widely adopted choice, STELAR is not as popular as ASTRAL, possibly due to its limitation in analyzing unrooted gene trees. Gene trees are usually estimated using time reversible mutation models which makes the root of the tree non-identifiable [20].

Unrooted gene trees are commonly transformed into rooted trees by incorporating an outgroup that places the root between the outgroup and the remaining taxa in the tree. Another approach involves introducing the assumption of a molecular clock. However, identifying a suitable outgroup proves to be challenging, and the use of a pre-specified outgroup may lead to biased root placement [24,25]. Therefore, in the absence of a molecular clock or a reliable outgroup, alternative techniques for rooting phylogenetic trees have been developed [26–31]. Despite the long history of thinking about tree rooting, there has been a lack of investigation into their impact in the context of estimating species trees using methods that rely on rooted gene trees.

In this study, we focus on triplet-based species tree estimating methods and investigate the robustness (or lack thereof) of these methods to variations in gene tree rooting. We report, on an extensive experimental study using a collection of simulated as well as empirical datasets, the performance of STELAR with gene trees rooted by different techniques. Furthermore, we identified different model conditions where STELAR with algorithmically rooted gene trees (i.e., rooted with methods other than outgroup rooting) outperformed not only STELAR paired with outgroup rooting but also the state-of-the-art ASTRAL. These results indicate the potential for finding appropriate roots for the gene trees that result in better species tree estimations than ASTRAL, and thus we believe opens up the avenue for further research into the impact of gene tree rooting on the performance of triplet-based species tree estimation methods.

## 2 Background and preliminaries

We now give a brief overview of various rooting techniques analyzed in this study, namely Mid Point (MP), Minimum Variance (MV), Minimal Ancestor Deviation (MAD), Root-digger (RD), outgroup (OG), and random rooting.

### 2.1 Rooting methods

Phylogenetic trees can be categorized as rooted or unrooted, each serving distinct purposes in evolutionary research. Rooted trees identify the last common ancestor, providing crucial insights into the directionality of evolution, whereas unrooted trees focus on the relationships among taxa without specifying evolutionary paths. Many methods, each with their own strengths and drawbacks, have been established for rooting unrooted phylogenetic trees.

The outgroup (OG) rooting method is the most commonly used technique for rooting phylogenetic trees. While this method generally provides better rooting accuracy when compared to other rooting methods [32], the main challenge lies in selecting an appropriate outgroup [33–38]. In cases where an appropriate outgroup is unknown, inferring a root is possible using a molecular clock [32,39]. A strict molecular clock assumes a constant rate of substitution across all lineages under consideration; a rather problematic assumption in cases where the ingroup taxa may be distantly related and, consequently, have varying rates of molecular evolution. Relaxed molecular clocks, while comparatively more robust [39], still struggle with accuracy when deviations from a clock-like rate are substantial [40].

Furthermore, there are rooting methods that take into account the distribution of branch lengths (and/or branch supports). Midpoint Rooting [41], Minimum Variance Rooting [42], and Minimal Ancestor Deviation [40] fall under this category. These methods also assume a clock-like behavior and, though they exhibit less dependence, their accuracy also decreases with deviations from this assumption.

Other methods that do not root using an outgroup include gene duplication-based rooting [26,43,44], indel-based rooting [27,45,46], rooting species trees using distributions of unrooted gene trees [28], probabilistic co-estimation of gene and species trees [29], rooting using a non-reversible Markov model with the help of multiple sequence alignments [30], and rooting species trees using the distribution of quintets induced by gene trees [31]. We note that rooting gene trees using gene duplication events can be viewed as a special case of outgroup rooting, where the paralogous copy plays the role of an outgroup.

Another class of methods using Bayesian time-tree inference [e.g., 47,48] provides rooting signal via probabilistic priors (e.g., birth-death [49,50] and coalescent models [51,52]). These priors favour branching-time configurations consistent with the assumed diversification or population processes, typically disfavoring root placements that imply implausibly long or highly asymmetric deep branches. However, because these approaches jointly estimate topology, branch lengths as divergence times, and root position under a molecular clock, they fundamentally reparameterise the input tree. They therefore fall outside the scope of this study, which focuses on *a posteriori* rooting of fixed, unrooted gene trees.

Among the wide range of methods available for rooting phylogenetic trees, some methods are designed to co-estimate rooted gene and species trees [9,29], while some others are specifically developed for rooting species trees [28,31]. Most other approaches, such as outgroup rooting and midpoint rooting, are general-purpose methods that can be applied to both gene trees and species trees. In this study, we explore the effect of the following methods on gene tree rooting: Outgroup (OG) rooting, Mid-Point (MP) rooting, Minimum Variance (MV) rooting, Minimum Ancestor Deviation (MAD) rooting, Root-Digger (RD), and Random (RAND) rooting. These methods were not restricted to only species trees and thus could be utilized to root gene trees. We discuss in details about these rooting methods in the subsequent sections.

#### 2.1.1 Outgroup rooting method (OG).

The outgroup method is a popular rooting approach in phylogenetics [32,53–56], often used to determine the evolutionary starting point of a tree. This method assumes that one or more taxa (the outgroup) are divergent from the main group being studied (the ingroup). The branch connecting the outgroup and ingroup sets the evolutionary baseline for the tree [57,58]. The outgroup method not only aids in understanding ingroup evolution but also helps in identifying unique features within ingroup sequences. However, selecting an appropriate outgroup is challenging [33–38], particularly for datasets with a large number of taxa where a consensus about the outgroup is lacking [59]. If an outgroup is too distantly related to the ingroups, it might adversely impact the rooting accuracy owing to a substantially different molecular evolution. Conversely, outgroups that are too closely related with the ingroups may fail to perform its functions as an appropriate outgroup.

Incorrect outgroup selection can also lead to long branch attraction (LBA), a phenomenon where distant outgroup taxa erroneously influence the tree due to their large divergence time or rapid evolution [55]. This results in artifactual or random rooting [60,61]. To mitigate LBA, several criteria, such as low substitution rate and phylogenetic proximity, are suggested for choosing outgroups, particularly in complex cases like arthropod classes [62]. Additionally, [25] recommends using multiple outgroup samples within the sister group to minimize LBA and enhance the robustness of the outgroup rooting method. [63] demonstrated that introducing just one additional taxon can significantly alter the resulting tree topology, impacting even the existing ingroup taxa. [64] further confirms that it is common for outgroups to influence ingroup topologies.

OGs can also be used as “true” roots, if the original root is known beforehand (as in the case in simulated datasets).

#### 2.1.2 Mid-point rooting method (MP).

Midpoint rooting in phylogenetics is a method where the root is placed at the midpoint between the two furthest tips of the tree [36,41]. This method works effectively if the tree exhibits constant rates of evolution, relying on the assumption of a molecular clock and homogenous evolution rates across branches [64]. Midpoint rooting is particularly suitable for balanced trees but has limitations, especially if the tree data is not clocklike or the topology is unbalanced. The midpoint rooting method usually displays an impressively high success rate, which is especially remarkable in the multiple outgroup, consistently rooted datasets (MOC) [54].

This technique is frequently used in studies where outgroups are not available, such as in viral genetics. An example is [65], who applied midpoint rooting to analyze the evolutionary relationships of SARS coronaviruses, focusing on genes encoding envelope matrix and nucleocapsid proteins. [66] rooted phylogenetic trees using Midpoint-rooting method, which helped in genome-based phylogeny and classification of prokaryotic viruses. Moreover, phylogenetic reconstruction was done in [67] using midpoint rooting technique. The appropriateness of midpoint rooting is supported by the results of the relative rate test by [68], indicating no rate heterogeneity among the coronavirus groups [65]. It is advised not to use MP as the default method and to only use it if the outgroup method is not applicable, e.g., due to a lack of a priori knowledge regarding the outgroup or LBA [54].

#### 2.1.3 Minimum variance rooting method (MV).

This section discusses the rooting method introduced by [42], which focuses on reducing the variance of root-to-tip distances in the phylogenetic tree. It is effective in cases where deviations from the strict molecular clock are random and can be implemented in a linear time algorithm, similar to the traditional midpoint rooting method.

The effectiveness of this method has been explored through extensive simulations, considering factors like gene tree estimation error, divergence from the clock, and outgroup distance to ingroups. [42] showed that MV rooting performs better or at least as well as midpoint rooting across various conditions, especially when the divergence from the clock is less and outgroup distance is smaller. [69] used this method to root phylogenies under Outgroup-free rooting.

#### 2.1.4 Root-digger rooting method: Search (RD) and exhaustive (RD-EX).

RootDigger, introduced by [30], is a tool that takes an unrooted phylogenetic tree and its corresponding Multiple Sequence Alignment (MSA), and outputs a rooted tree. It uses a non-reversible Markov model to determine the most likely root location on the tree and infer confidence values for each potential root placement. It attempts to address the limitations of existing methods like molecular clock analysis (including midpoint rooting) and outgroup rooting. RootDigger circumvents the computationally demanding task of inferring a tree with a non-reversible model by using a reversible model for fast tree inference and then applying a non-reversible model solely for rooting the tree in a final step.

RootDigger was used by [70] for Phylogenetic analysis of bestrhodopsins. Here traditional outgroup method failed due to significant evolutionary distance. Alternative approaches such as RootDigger, which uses non-reversible Markov models, was applied to improve rooting accuracy.

RootDigger operates in two modes: Search and Exhaustive. The Search mode quickly identifies the most likely root using heuristics, while the Exhaustive mode thoroughly evaluates the likelihood of placing the root in every branch of the tree, reporting the Likelihood Weight Ratio for each branch. We considered both the search (RD) and exhaustive (RD-EX) modes for our study.

#### 2.1.5 Minimal ancestor deviation rooting method (MAD).

The Minimal Ancestor Deviation (MAD) rooting method is a phylogenetic technique designed to identify the root of an unrooted tree by minimizing deviations from the strict molecular clock hypothesis [40]. While strict ultrametricity rarely holds in practice, the midpoint criterion asserts that the middle of the path between two operational taxonomic units (OTUs) should coincide with their last common ancestor (LCA). The MAD algorithm evaluates this criterion by considering each branch of the tree as a potential root and then calculating the mean relative deviation from the molecular clock expectation for all OTU pairs. The branch that minimizes the deviation is considered the best candidate for the root. The deviation between the observed and expected distances from a putative ancestor node to any two OTUs is quantified by comparing the distance of each OTU to the ancestor with half the distance between the two OTUs. This method tries to ensure that the root placement is the one that best aligns with the expected molecular clock-based distances.

## 3 Experimental studies

### 3.1 Datasets

We studied a collection of previously used simulated and biological datasets to evaluate the impact of various rooting techniques on the triplet-based summary method STELAR. We also compared STELAR (with various rooting techniques) with ASTRAL, the leading coalescent-based summary method, which maximizes quartet-consistency and thus does not require rooted gene trees.

#### 3.1.1 Simulated dataset.

We used two biologically-based simulated datasets from [71], which were generated based on species trees estimated by MP-EST [14] on avian (48 taxa) and mammalian (37 taxa) datasets from [8] and [72], respectively. For the avian simulations, gene sequence lengths were varied (250 bp, 500 bp, 1000 bp, and 1500 bp), while in the mammalian simulations, we examined sequence lengths of 250 bp, 500 bp, and 1000 bp to assess the impact of phylogenetic signal. In both simulations, we modeled three levels of incomplete lineage sorting (ILS) – low [2X], moderate [1X] and high [0.5X] – by scaling internal branch lengths in the species tree and also explored varying gene counts.

We also used a 15-taxon dataset from [73] with a caterpillar-like (pectinate or ladder-like) model species tree, featuring 12 consecutive short internal branches (0.1 coalescent units) – creating conditions for high levels of ILS. Ultrametric gene trees were simulated along this tree under a multi-species coalescent model, adhering to a strict molecular clock without branch length transformations. Sequence data were generated for each gene tree, and four model conditions were constructed by using gene sequence lengths of 100 or 1000 sites and using either 100 or 1000 genes.

For 11 taxa, we specifically analyzed the regions exhibiting strong incomplete lineage sorting (ILS). The number of genes analyzed varied incrementally, with counts set at 5, 15, 25, 50, and 100 genes.

The simulated datasets we studied varied in many respects (number of genes, sequence length per gene, whether the sequence evolution is ultrametric or not, and the ILS level). Thus, as summarized in Table 1, they represent a wide range of model conditions on which we evaluated the impact of various rooting techniques on the performance of the triplet-based species tree estimation method STELAR. To clearly distinguish them from empirical datasets, we refer to a simulated dataset of *n* taxa using the notation *n*tax-sim. We also analyzed two relatively larger datasets containing 200 and 500 taxa [22]. For both these datasets, we analyzed tree lengths 2 M generations with speciation rates 1e-6 per generation. SimPhy [74] was used to simulate species trees [22] based on the Yule process, defined by parameters such as the number of taxa, the maximum tree length, and the speciation rate, which together specify a model condition. The tree length impacts the amount of ILS, with lower length resulting in shorter branches, and therefore higher levels of ILS. We used medium tree length (2 M) that indicates moderate level ILS. 10 replicates of each dataset (200 taxa, 500 taxa) were analyzed, with each replicate comprising 1,000 gene trees. These datasets were simulated according to the multi-species coalescent model with the population size fixed to 200000 [22]. The detailed simulation procedures for the datasets are described in Section 1 of S1 Appendix.

**Table 1:** Properties of the simulated datasets. The level of ILS is represented by the average topological distance between the true gene trees and the true species tree. All datasets were simulated under the multispecies coalescent (MSC) model. Gene sequences were simulated and gene trees were estimated under time-reversible substitution models. RAxML [75] was used to infer gene trees.

Dataset	ILS level	# genes	# sites	# rep.	Model	Time Rev.	Reference
11tax-sim	85%	5 - 100	2000	20	MSC	Yes	[76]
15tax-sim	82%	100 - 1000	100 - 1000	10	MSC	Yes	[73]
37tax-sim	18%, 32%, 54%	200 - 800	500 -1000	20	MSC	Yes	[71]
48tax-sim	35%, 47%, 59%	25 - 1000	250 -1000	20	MSC	Yes	[71]
200tax-sim	10% - 70%	1000	300 - 1500	10	MSC	Yes	[22]
500tax-sim	10% - 70%	1000	300 - 1500	10	MSC	Yes	[22]

#### 3.1.2 Empirical dataset.

**Angiosperm dataset** We used the angiosperm dataset of 310 nuclear genes analyzed in [22,77]. The nuclear gene taxon sampling included 42 species representing all major angiosperm clades (35 families and 28 orders [78]). Three gymnosperms (*Picea glauca* [Moench] Voss, *Pinus taeda* L., and *Zamia vazquezii* D.W. Stev., Sabato & De Luca) and one lycophyte (*Selaginella moellendorffii* Hieron.) were included as outgroups. These three gymnosperms span the crown node of extant gymnosperms [79].

**Amniota dataset** We used the Amniota dataset from [80] containing 16 species and 248 genes. We analyzed the nucleotide (NT) part of the dataset where *Protopterus annectens* was used as outgroup.

**Mammalian dataset** We used the mammalian dataset from [72] which contains 447 genes across 37 mammals, after removing 21 mislabeled genes (confirmed by the authors), and two other outlier genes. *Gallus* is the outgroup in this dataset.

### 3.2 Methods compared

We investigated the performance of different rooting techniques paired with STELAR, a statistically consistent triplet-based species tree estimation method. Thus our experimental pipeline contains the following steps: 1) take a set of unrooted gene trees, 2) root them using an appropriate rooting method of our choice, and finally, 3) infer the rooted species tree using a triplet-based and statistically consistent species tree estimation method (STELAR [17]), with a detailed workflow outlined in Fig 1. As discussed in detail in Section 2.1, we used the following rooting methods: Outgroup Rooting (OG), Midpoint Rooting (MP) [41], Minimum Variance rooting (MV) [42], Minimum Ancestor Deviation rooting (MAD) [40], RootDigger(RD and RD-EX) [30], Random rooting (RAND). Bayesian time-tree rooting methods were not included in this study, as they jointly estimate topology, branch lengths, and root position under a clock model, thereby modifying the input tree rather than performing post hoc rooting. We refer to STELAR paired with a particular rooting technique using the convention STELAR–⟨quartet-generation-technique⟩ (e.g., STELAR-OG, STELAR-MP, etc.). We also compared STELAR paired with different rooting techniques with ASTRAL (the latest ASTRAL-III version [23]), the leading species tree estimation method which maximizes quartet consistency. In addition, to assess whether the observed effects generalize beyond STELAR, we performed supplementary experiments using SuperTriplets [81], another triplet-based species tree estimation method.

**Fig 1:**
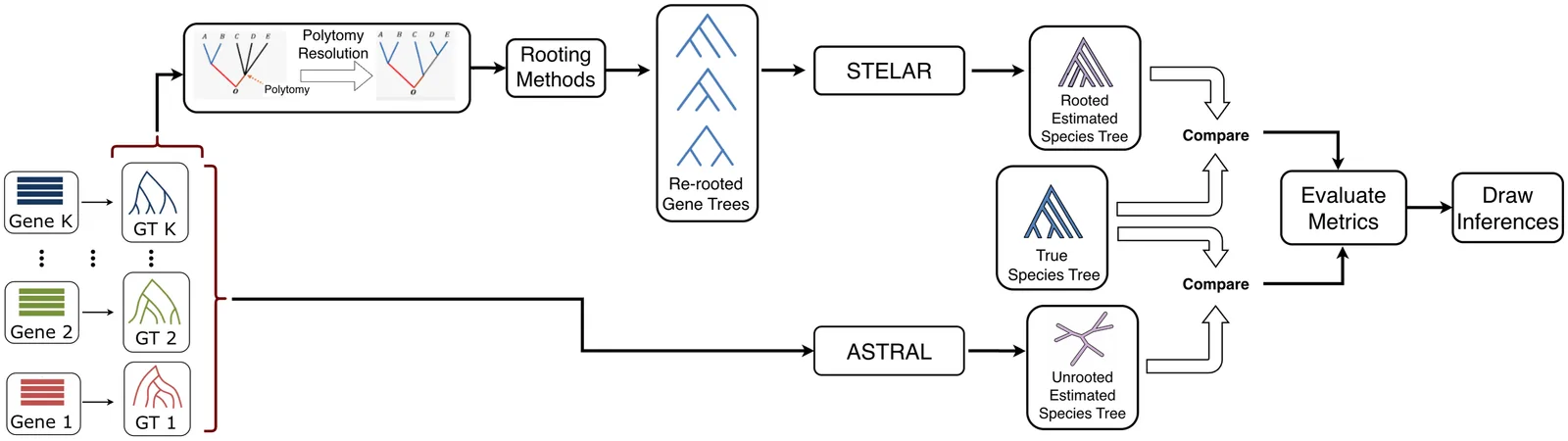
Overview of the experimental workflow for evaluating the impact of gene tree rooting on species tree estimation. We start with a set of gene sequences and the corresponding gene trees (estimated using maximum likelihood based method). After preprocessing steps (such as polytomy resolution), these gene trees are rerooted using different rooting methods (e.g., OG, MP, MV, MAD, etc.). Afterwards, STELAR uses these sets of rerooted gene trees to estimate corresponding species trees. In parallel, ASTRAL is used to infer species trees directly from the unrooted gene trees. The estimated species trees are compared to the true species tree via the Robinson-Foulds (RF) distance, and other metrics such as triplet and quartet scores are also evaluated. Finally, these results are analyzed to draw inferences. Additional details about the simulated dataset generation process are described in Section 1 of S1 Appendix.

### 3.3 Evaluation metrics

We compared the estimated trees (on simulated datasets) with the model species tree using normalized Robinson-Foulds (RF) distance [82], which is a widely used metric to measure tree error. The RF distance between two trees is the sum of the bipartitions (splits) induced by one tree but not by the other, and vice versa.

We also compared the triplet and quartet scores of candidate species trees. Triplet score of a rooted species tree *T* with respect to a particular set 𝒢 of rooted gene trees is defined as the number of triplets induced by the gene trees that the candidate tree *T* is consistent with. Maximizing triplet score is a statistically consistent criterion for estimating species trees [17]. We use the term true triplet score (TTS) when we compute the triplet score with respect to true gene trees (no estimation error). We similarly define and use the metrics quartet score and true quartet score, which consider quartet consistency instead. When reporting these scores in tables, we normalize triplet and quartet scores by the total number of possible induced structures across all gene trees, i.e., |𝒢|×(n3) for triplets and |𝒢|×(n4) for quartets.

To assess the correctness of the root placements inferred by various techniques, we computed the topological distance between the inferred root and the true root [30]. This is defined as the number of nodes in the path from the inferred/estimated root to a predefined true root. This distance quantifies the error in root placement without any scaling or normalization [30] based on tree size. This metric was introduced to quantify errors in root placement, addressing the limitations of the binary “percentage of correct rooting” measure, which failed to capture such nuances. It is worth noting that, since each dataset analyzed contains a single outgroup, we define a rooting as “correct” if the inferred root separates the outgroup from the ingroup, regardless of whether the inferred unrooted topology is otherwise correct.

## 4 Results and discussion

### 4.1 Simulated datasets

#### 4.1.1 37tax-sim.

Fig 2 shows the performance of STELAR with gene trees rooted by different methods and ASTRAL on the 37tax-sim dataset under various model conditions. One of the key observations from the 37tax-sim dataset is the robustness of the triplet-based species tree estimation method, STELAR, to gene trees rooted by various techniques. STELAR paired with most rooting methods, including MP, MV, MAD, and RD-EX, resulted in competitive species trees with no statistically significant differences (as seen in Fig 2). Surprisingly, while random rooting (RAND) performed worse in many instances, it still remained competitive under certain model conditions, such as 2X ILS (Fig 2c), 1000 bp, and true gene trees (Fig 2b). Despite yielding comparable accuracy in the 2x ILS model condition (Fig 2c), STELAR performed substantially worse in all other model conditions when working with gene trees rooted by RD. On the other hand, RD-EX rooting performed notably better in most model conditions of 37tax-sim. It is worth noting, because RD is designed for non-reversible substitution models, simulations generated under time-reversible models (including 37tax-sim) may not fully reflect its intended strengths. Nevertheless, RD-EX also underperformed on the empirical datasets (Section 4.2.1).

**Fig 2:**
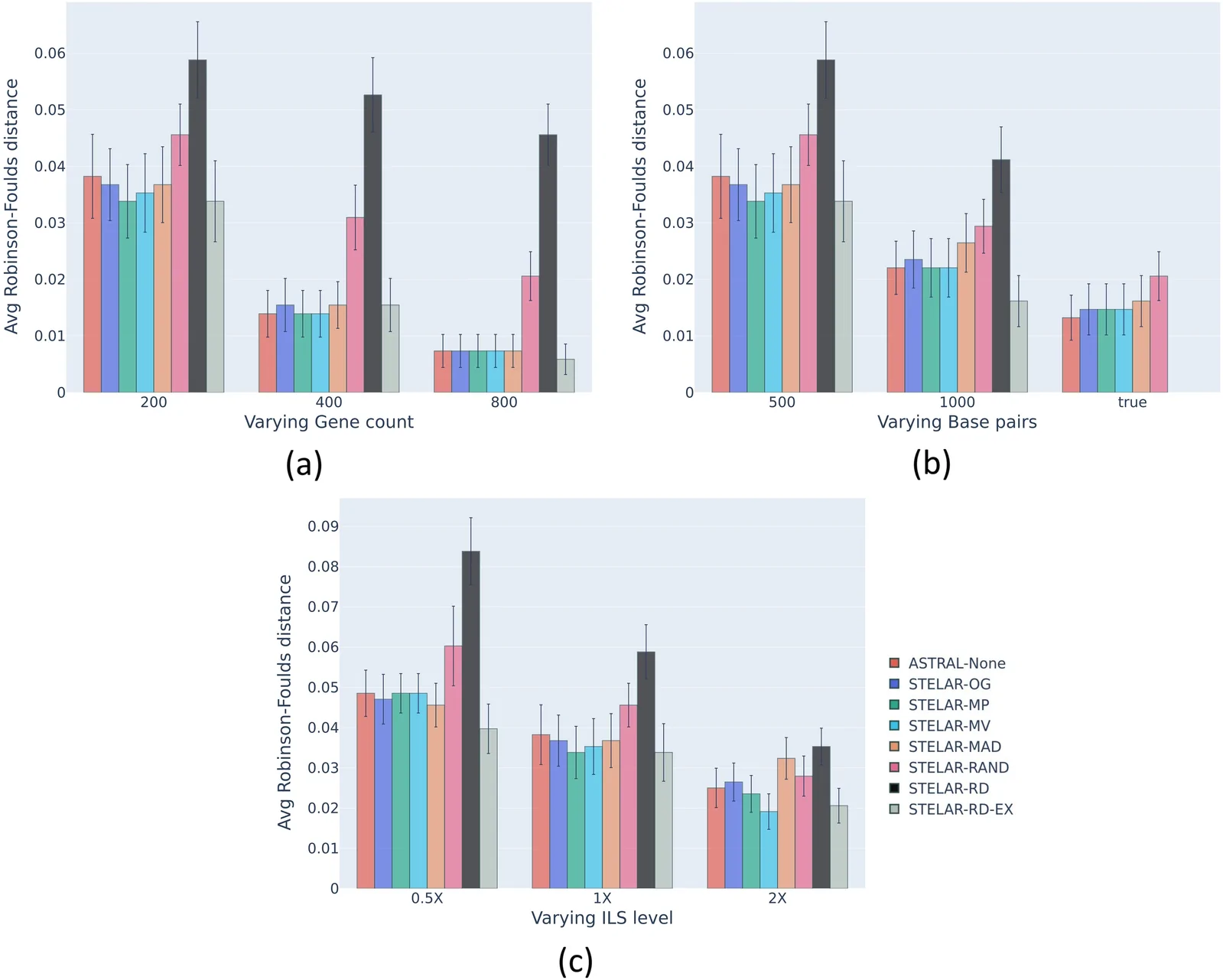
Comparison of Average Robinson-Foulds distance with standard error bars over 20 replicates for ASTRAL and STELAR with different rooting methods for the 37tax-sim mammalian dataset. **(a)** We fixed the ILS at moderate (1X) and the base pairs at 500 bp, and varied gene counts between 200, 400 and 800. RD could not be run on 400 genes, since the gene sequences corresponding to the gene trees were not available. **(b)** We varied the sequence length, and consequently the gene tree estimation error, between 500, 1000 and true-gt while keeping moderate ILS (1X) and 200 gene trees. **(c)** We set the gene trees at 200, the sequence length at 500 bp, and varied the ILS between high (0.5X), moderate (1X) and low (2X).

Interestingly, STELAR sometimes yielded more accurate species trees with rootings other than the known/true OG. There were model conditions where gene trees rooted using MP, MV, MAD, and RD-EX resulted in superior species trees compared to OG and even ASTRAL, albeit these differences were not statistically significant. Such cases include MAD in 0.5X and 1X ILS cases, MP, MV in the 1X-200gene-500 bp and 2X-200gene-500 bp model conditions, and RD-EX in all conditions except 1x-400gene-500 bp.

The general patterns were consistent with our expectations: for all methods, the species tree estimation accuracy was improved by increasing the number of genes and sequence lengths (i.e., decreasing the gene tree estimation error), but was reduced by increasing the amount of gene tree discordance (i.e., the amount of ILS). In general, the choice of rooting techniques becomes less impactful in “easier” model conditions (e.g., higher gene count and more base pairs), with STELAR with MP, MV, OG, and MAD all producing the same or similar tree accuracy (e.g., under conditions with 800 genes and true gene trees).

Next, we compared the relative performance of different rooting techniques in terms of species tree accuracy. RD-EX, MP, and MV perform notably well on this dataset, especially in “unfavorable” model conditions with fewer genes, shorter gene sequences, and higher ILS. MP and RD-EX matched or outperformed most other methods, including ASTRAL, across various model conditions. RD-EX especially performed well in conditions with high (0.5X) ILS and high basepair count (1000 bp). Similarly, MV performed on par with other methods, including ASTRAL, and showed significant improvements under low ILS (2X) conditions.

**Table 2:** Recovery of major clades in the Amniota biological dataset. Each grid cell denotes whether an expected clade was recovered (green) or not recovered (white) by a given method. The final row summarizes the total number of clades not recovered per method.

	Clade	ASTRAL	MAD	MP	MV	OG	RAND	RD	RDEX
1	Turtles *(((Chelonoidis, Emys), Caretta), Phrynops)*								
2	Crocodiles *(Caiman, Alligator)*								
3	Birds *(Gallus, Taeniopygia)*								
4	Archosaurs *((Crocodiles), (Birds))*								
5	Toxicofera *(Anolis, Python)*								
6	Squamates *(Podarcis, Anolis, Python)*								
7	Mammals *(Monodelphis, Homo, Ornithorhynchus)*								
8	Ingroup (*Amniota*)								
	**# Missing Clades**	**0**	**2**	**2**	**1**	**0**	**0**	**3**	**3**

Although MAD achieved one of the best species tree accuracies at high ILS (0.5x), its performance remained largely unaffected by varying levels of ILS. This unresponsiveness of MAD was also observed toward varying gene tree estimation errors (controlled by sequence lengths). However, like other methods, MAD’s performance improved with an increasing number of genes.

To assess whether these observations generalized beyond STELAR, we also conducted experiments with the triplet based species tree estimation method SuperTriplets [81] on this 37tax-sim dataset. While we observed reduced performance of RD-EX under several model conditions (detailed in Section 3.1 of S1 Appendix), the overall trends in RF scores remained qualitatively similar (Fig A of S1 Appendix). This suggests that the impact of gene tree rooting on species tree inference is not specific to STELAR.

**Table 3:** Recovery of key topological relationships in the Angiosperm Biological Dataset. Grids show the presence (green) or absence (white) of expected topological relationships recovered by each method. Rows focus on basal angiosperm relationships (not necessarily clades), as these represent the most phylogenetically contentious splits in the dataset. Relationships among eudicots and monocots were consistently recovered across all methods. Numbers of expected relationships not recovered are listed below each column.

	Relationship	ASTRAL	MAD	MP	MV	OG
1	Clade of Angiosperms without *Nuphar* and *Amborella*					
2	*Nuphar* as a sister to Angiosperms except *Amborella*					
3	*Amborella* as a sister to all other Angiosperms					
4	Outgroups *(Selaginella, (Zamia, (Pinus, Picea)))*					
	**# Missing Relationships**	**0**	**2**	**4**	**4**	**4**

To further investigate why RD-EX, MP, MV, and occasionally MAD yielded better RF scores than OG, and why RD performed poorly, we conducted a series of experiments examining triplet and quartet scores, as well as the correctness of the roots of the gene trees and species trees inferred by various techniques. First, we compared the triplet scores (Table 4) and quartet scores (Table 5) of the estimated species trees. As expected, due to the statistical consistency property of the triplet score, in almost all cases involving STELAR, a lower RF score corresponds to a higher triplet score. For instance, under high ILS conditions (0.5X), MAD achieved the highest triplet score and the lowest RF score. Interestingly, OG, which is expected to yield the highest triplet score when the outgroup is known for simulated datasets, did not perform as expected under certain model conditions (e.g., 1X-200gt-1000 bp and 1X-400gt-500 bp). In these scenarios, STELAR using gene trees rooted by OG resulted in marginally lower triplet scores and higher RF scores compared to STELAR with gene trees rooted by MP and MV. There were a few exceptions to this trend, such as in the 1X-200gt-500 bp and 2X-200gt-500 bp conditions, where OG achieved higher triplet scores despite MP and MV performing better in terms of RF scores. In some cases (e.g., 0.5X-200gt-500 bp and 1X-200gt-1000 bp) where RD-EX achieved the best RF scores, it displayed slightly lower triplet scores - an exceptional trend that can be attributed to higher variance in the RF scores that were averaged.

**Table 4:** We report the Average Triplet Scores (TS) of the species trees inferred by ASTRAL and by STELAR using gene trees rooted with different techniques (e.g., MP, MV, etc.). In each row (model condition), the best value is highlighted in boldface. For readability and comparability across datasets with different numbers of taxa and gene trees, TS values are normalized by the total number of possible triplets (i.e., (n3) multiplied by the number of gene trees) and reported to four decimal places. Boldface indicates the largest value in each row based on the underlying unrounded normalized scores; ties may appear due to rounding. Missing entries under RD and RD-EX indicate that corresponding MSAs were unavailable, preventing these methods from being run. Similarly, empty cells under MAD reflect the absence of branch lengths and support values.

Dataset	Model Cond^*n*^	MAD	MP	MV	OG	RAND	RD	RDEX
**11tax-sim**	strong-5		.7777	.7777	**.9232**	.6535		
	strong-15		.7800	.7800	**.9257**	.7479		
	strong-25		.7754	.7754	**.9209**	.7634		
	strong-50		.7745	.7745	**.9200**	.7700		
	strong-100		.7736	.7736	**.9191**	.7718		
**15tax-sim**	100gt-100bp	.3788	**.3889**	**.3889**	**.3889**	.3617	.3885	.3707
	100gt-1000bp	.4420	**.4460**	**.4460**	**.4460**	.4074	.4470	.4353
	100gt-true		.5266	.4881	**.7214**	.4368		
	1000gt-100bp	.3772	**.3867**	**.3867**	**.3867**	.3599	.3866	.3691
	1000gt-1000bp	.4368	.4387	.4387	.4387	.4007	**.4435**	.4282
	1000gt-true		.5298	.4805	**.7201**	.4480		
**37tax-sim**	.5X-200-500	**.6367**	.6367	.6365	.6366	.5625	.5426	.6318
	1X-200-1000	.7455	**.7455**	**.7455**	.7455	.6186	.6049	.7324
	1X-200-500	.7088	.7090	.7089	**.7094**	.6131	.6000	.7040
	1X-200-true	**.9078**	**.9078**	**.9078**	**.9078**	.6428		
	1X-400-1000	.7447	**.7447**	**.7447**	**.7447**	.6198	.6197	.7320
	1X-400-500	.7087	**.7087**	**.7087**	.7087	.6131	.6130	.7029
	1X-400-true	**.9075**	**.9075**	**.9075**	**.9075**	.6429		
	1X-800-1000	**.7440**	**.7440**	**.7440**	**.7440**	.6203	.6202	.7314
	1X-800-500	**.7104**	**.7104**	**.7104**	**.7104**	.6131	.6129	.7040
	1X-800-true	.8815	**.9080**	**.9080**	**.9080**	.6432		
	2X-200-500	.7419	.7431	.7426	**.7432**	.6364	.6223	.7372
**48tax-sim**	.5X-1000-500	.5492	.5987	.5536	**.6133**	.4586	.4348	.6041
	1X-100-500	.4162	.6175	.5112	**.6807**	.4929	.4827	.6063
	1X-1000-500	.4396	.6579	.5078	**.6779**	.4913	.4723	.6432
	1X-200-500	.4337	.6396	.5047	**.6798**	.4950	.4770	.6378
	1X-25-500	.4555	.6255	.5086	**.6892**	.4972	.4919	.5867
	1X-50-500	.4226	.6290	.5240	**.6841**	.4955	.4866	.5982
	1X-500-500	.4394	.6585	.5047	**.6776**	.4990	.4742	.6395
	2X-1000-500	.4027	.6627	.4592	**.6891**	.5174	.4958	.6347
**200tax-sim**	Estimated	.5751	.5827	.5709	.5784	**.5983**		
**Amniota**	Nucleotide	.1067	.1106	.1075	.1075	**.1287**	.1081	.1114
**Angiosp** ^***m***^	Nuclear	**.2581**	.2551	.2551	.2551	.1844		

**Table 5:** We calculate the Average Quartet Scores (QS) of the estimated species trees inferred by ASTRAL and STELAR paired with different rooting techniques. Similar to Table 4, the QS values are normalized by the total number of possible quartets ((n4) times the number of gene trees) and reported to four decimal places. We highlight the largest normalized value in each row (before rounding) by boldface; ties may appear post-rounding.

Dataset	Model Cond^*n*^	ASTRAL	STELAR						
			MAD	MP	MV	OG	RAND	RD	RDEX
**11tax-sim**	strong-5	**.8227**		.8224	.8224	.8214	.7993		
	strong-15	**.8219**		.8215	.8215	.8211	.8012		
	strong-25	**.8116**		.8115	.8115	.8115	.7995		
	strong-50	**.8111**		.8111	.8111	.8109	.7994		
	strong-100	**.8093**		**.8093**	**.8093**	.8092	.8041		
**15tax-sim**	100gt-100bp	**.5123**	.5105	.5075	.5075	.5075	.5039	.5095	.5000
	100gt-1000bp	**.6020**	.6015	.6008	.6008	.6008	.5906	.5995	.5959
	100gt-true	**.6200**		.6192	.6196	**.6200**	.6103		
	1000gt-100bp	**.5082**	.5066	.5015	.5015	.5015	.5003	.5018	.5035
	1000gt-1000bp	**.5993**	.5992	.5989	.5989	.5989	.5945	.5973	.5947
	1000gt-true	**.6185**		**.6185**	**.6185**	**.6185**	.6106		
**37tax-sim**	.5X-200-500	**.7576**	.7576	.7576	.7576	.7576	.7570	.7567	.7575
	1X-200-1000	**.8772**	.8772	.8772	.8772	.8772	.8768	.8766	.8771
	1X-200-500	**.8533**	.8532	.8532	.8532	.8533	.8527	.8523	.8532
	1X-200-true	**.8893**	.8893	.8893	.8893	.8893	.8889		
	1X-400-1000	**.8767**	.8767	**.8767**	**.8767**	**.8767**	.8762	.8761	.8766
	1X-400-500	**.8528**	.8528	.8528	.8528	.8528	.8524	.8518	.8528
	1X-400-true	**.8891**	**.8891**	**.8891**	**.8891**	**.8891**	.8889		
	1X-800-1000	**.8771**	**.8771**	**.8771**	**.8771**	**.8771**	.8769	.8765	**.8771**
	1X-800-500	**.8535**	**.8535**	**.8535**	**.8535**	**.8535**	.8533	.8527	.8535
	1X-800-true	**.8637**	**.8637**	**.8637**	**.8637**	**.8637**	.8635		
	2X-200-500	**.9045**	.9043	.9045	.9045	.9045	.9042	.9039	.9045
**48tax-sim**	.5X-1000-500	**.5683**	.5328	.5620	.5346	.5673	.5632	.5640	.5669
	1X-100-500	**.6364**	.6180	.6238	.6149	.6331	.6274	.6259	.6272
	1X-1000-500	**.6323**	.6130	.6259	.5907	.6317	.6270	.6282	.6272
	1X-200-500	**.6350**	.6158	.6264	.6132	.6332	.6275	.6263	.6287
	1X-25-500	**.6476**	.6276	.6274	.6256	.6405	.6306	.6280	.6359
	1X-50-500	**.6408**	.6234	.6227	.6178	.6354	.6293	.6254	.6289
	1X-500-500	**.6329**	.6137	.6260	.6017	.6319	.6268	.6281	.6276
	2X-1000-500	**.6765**	.6638	.6676	.6518	.6761	.6689	.6719	.6694
**200tax-sim**	estimated	**.7362**	.7349	.7335	.7346	.7361	.7326		
**Amniota**	Nucleotide	**.2169**	.2165	.2165	.2169	**.2169**	**.2169**	.2062	.2086
**Angiosp** ^***m***^	Nuclear	**.2284**	.2167	.2162	.2162	.2162	.2216		

We also looked at the quartet scores of the trees estimated by ASTRAL and the STELAR variants. ASTRAL often failed to achieve the best RF scores, even though it always had the highest quartet scores (see 0.5x-200gt-500 bp, 1x-200gt-500 bp, 2x-200gt-500 bp model conditions). Similar to triplet-scores, when STELAR-MP and STELAR-MV outperformed STELAR-OG in species tree accuracy (e.g., 1X-200gt-1000 bp and 1X-400gt-500 bp), they tended to achieve higher quartet scores. However, as with triplet scores, there were exceptions to this trend in some model conditions such as 0.5x-200gt-500 bp and 2X-200gt-500 bp, and for the few aforementioned cases of RD-EX.

Next, we examined the percentage of correctly rooted gene trees among the inputs to STELAR for each rooting method. This simulated dataset is rooted using the outgroup (Chicken, Turkey). Aside from the obvious 100% accuracy from OG, we see MV consistently reaching the second best, closely followed by MP and then MAD (Table 6). RAND, RD, and RD-EX scored poorly in this metric. To further evaluate gene tree rooting accuracy for the rooting methods, we calculated the average topological distance between the true root and inferred root for the gene trees, in Table 7. This analysis supports the trend seen above: by definition, OG scores 0, indicating perfect accuracy, while MV, MP, and MAD demonstrate progressively increasing topological distances, reflecting their respective accuracies in inferring the root position. RD-EX, RAND, and RD give higher distances on average, corroborating their low percentage of correct rootings.

**Table 6:** We evaluate the ability of rooting techniques to predict the true root by calculating the Percentage of correctly rooted gene trees for each model condition. Boldface indicates best value in each row.

Dataset	Model Cond^*n*^	MAD	MP	MV	OG	RAND	RD	RDEX
**11tax-sim**	strong-5		0.0	0.0	**100.0**	11.0	0.0	
	strong-15		0.0	0.0	**100.0**	8.9	0.0	
	strong-25		0.0	0.0	**100.0**	9.0	0.0	
	strong-50		0.0	0.0	**100.0**	9.9	0.0	
	strong-100		0.0	0.0	**100.0**	9.2	0.0	
**15tax-sim**	100gt-100bp	19.5	**100.0**	**100.0**	**100.0**	7.0	5.9	3.8
	100gt-1000bp	85.4	**100.0**	**100.0**	**100.0**	7.7	0.0	6.0
	100gt-true	0.0	0.0	0.0	**100.0**	6.5		
	1000gt-100bp	19.6	**100.0**	**100.0**	**100.0**	5.7	0.5	3.9
	1000gt-1000bp	83.8	**100.0**	**100.0**	**100.0**	5.9	0.0	5.7
	1000gt-true	0.0	0.0	0.0	**100.0**	6.1		
**37tax-sim**	0.5X-200-500	60.4	70.8	79.2	**100.0**	2.4	0.1	3.6
	1X-200-1000	78.8	81.4	86.9	**100.0**	2.4	0.1	5.0
	1X-200-500	66.9	77.6	85.4	**100.0**	2.5	0.0	5.5
	1X-400-1000	79.3	81.3	87.0	**100.0**	2.4	0.0	5.3
	1X-400-500	64.6	74.3	81.1	**100.0**	2.3	0.0	5.0
	1X-800-1000	78.5	80.9	86.6	**100.0**	2.5	0.0	5.1
	1X-800-500	67.5	78.2	85.3	**100.0**	2.3	0.0	5.3
	2X-200-500	71.6	80.8	87.1	**100.0**	2.8	0.1	6.4
**48tax-sim**	0.5X-1000-500	7.0	0.6	6.6	**100.0**	1.7	0.0	0.2
	1X-100-500	6.2	1.0	7.2	**100.0**	1.8	0.0	0.0
	1X-1000-500	5.2	0.8	6.1	**100.0**	1.6	0.0	0.0
	1X-200-500	5.7	0.9	6.9	**100.0**	1.6	0.0	0.0
	1X-25-500	7.0	0.8	8.4	**100.0**	2.2	0.0	0.0
	1X-50-500	6.3	1.2	7.6	**100.0**	2.2	0.0	0.0
	1X-500-500	5.3	0.7	6.2	**100.0**	1.8	0.0	0.0
	2X-1000-500	3.6	0.8	5.0	**100.0**	1.7	0.0	0.0
**200tax-sim**	estimated	6.6	9.3	11.2	**100.0**	0.0		
**500tax-sim**	true	25.3	0.0	0.0	**100.0**	0.0		
	estimated	7.4	12.5	16.7	**100.0**	0.0		

**Table 7:** To better evaluate the accuracy of rooting methods, we report the Average Topological Distance between true and estimated roots of gene trees for each rooting method under our model conditions. Bold values indicate the best entry in a row.

Dataset	Model Cond^*n*^	MAD	MP	MV	OG	RAND	RD	RDEX
**11tax-sim**	strong-5		1.180	1.330	**0.000**	3.340		
	strong-15		1.200	1.353	**0.000**	3.470		
	strong-25		1.182	1.344	**0.000**	3.420		
	strong-50		1.192	1.339	**0.000**	3.392		
	strong-100		1.199	1.347	**0.000**	3.475		
**15tax-sim**	100gt-100bp	3.536	**0.000**	**0.000**	**0.000**	5.253	3.685	5.343
	100gt-1000bp	0.763	**0.000**	**0.000**	**0.000**	5.031	3.372	4.769
	100gt-true		2.948	3.159	**0.000**	5.048		
	1000gt-100bp	3.516	0.001	0.007	**0.000**	5.329	3.738	5.271
	1000gt-1000bp	0.820	**0.000**	**0.000**	**0.000**	5.130	3.436	4.814
	1000gt-true		2.916	3.105	**0.000**	4.958		
**37tax-sim**	0.5X-200-500	1.423	0.634	0.547	**0.000**	7.606	8.085	4.378
	1X-200-1000	0.604	0.387	0.282	**0.000**	7.650	8.072	4.187
	1X-200-500	1.374	0.557	0.440	**0.000**	7.745	8.149	4.221
	1X-400-1000	0.602	0.391	0.282	**0.000**	7.616	8.094	4.172
	1X-400-500	1.341	0.551	0.444	**0.000**	7.649	8.168	4.224
	1X-800-1000	0.614	0.405	0.297	**0.000**	7.574	8.108	4.176
	1X-800-500	1.348	0.563	0.454	**0.000**	7.702	8.172	4.254
	2X-200-500	1.298	0.555	0.443	**0.000**	7.689	8.107	4.183
**48tax-sim**	0.5X-1000-500	5.555	5.075	5.677	**0.000**	9.026	9.324	4.746
	1X-100-500	7.554	5.700	6.815	**0.000**	9.267	9.514	7.560
	1X-1000-500	7.523	5.650	6.861	**0.000**	9.247	9.538	7.563
	1X-200-500	7.497	5.637	6.809	**0.000**	9.187	9.472	7.516
	1X-25-500	7.034	5.386	6.402	**0.000**	9.158	9.304	7.452
	1X-50-500	7.301	5.449	6.643	**0.000**	9.116	9.391	7.501
	1X-500-500	7.495	5.614	6.832	**0.000**	9.121	9.545	7.548
	2X-1000-500	8.599	6.047	7.496	**0.000**	9.364	9.830	7.925
**200tax-sim**	estimated	4.684	4.740	4.760	**0.000**	11.091		
**500tax-sim**	true	2.632	2.510	2.326	**0.000**	11.739		
	estimated	5.612	5.917	5.875	**0.000**	14.150		

Despite the fact that MP and MV did not achieve the same level of rooting accuracy as OG, as previously noted, the gene trees rooted by MP and MV produced species trees that were equally good or even better than those inferred using OG. This indicates the robustness of triplet-based methods in species tree estimation from gene trees rooted using different techniques under certain model conditions. RAND, as expected, failed to recover the correct root in most cases (around 98% of the time). Surprisingly, RD was even worse than random rooting (RAND), almost never recovering the correct root. This poor rooting accuracy of the gene trees rooted by RD likely explains the poor performance of STELAR-RD. STELAR-RD-EX, despite performing well in terms of RF scores in most cases, yielded low gene-tree rooting accuracy. Such a trend is better analyzed in the next dataset.

Finally, we examined the root of the estimated species trees. Unlike the quartet-based species tree estimation method ASTRAL, triplet-based methods work with rooted gene trees and infer a rooted species tree that maximizes the triplet score, making the rooting implied by the output of STELAR meaningful. We therefore analyzed the percentage of correct rooting in the inferred species trees as well as the average topological distance between the inferred species tree roots and the true root, as shown in Tables 8 and 9 respectively. Interestingly, despite the previously noted differences in RF scores, triplet scores, and quartet scores, all triplet-based methods except for STELAR-RD, STELAR-RD-EX, and STELAR-RAND correctly predicted the rooting of the species tree across all model conditions. Moreover, the topological distances between the roots inferred by RD-EX, RAND, and RD variants of STELAR and the true roots were not substantially large. These findings highlight the robustness of the triplet-based species tree estimation method STELAR in accurately inferring the root of the estimated species trees, even when the roots of the input gene trees differ.

**Table 8:** To evaluate STELAR’s ability to infer species tree roots, we calculate the Percentage of correctly rooted species trees inferred from gene trees rerooted by different rooting techniques. The best value from each row is highlighted in boldface.

Dataset	Model Cond^*n*^	MAD	MP	MV	OG	RAND	RD	RDEX
**11tax-sim**	strong-5		0.0	0.0	**100.0**	0.0	0.0	
	strong-15		0.0	0.0	**100.0**	0.0	0.0	
	strong-25		0.0	0.0	**100.0**	0.0	0.0	
	strong-50		0.0	0.0	**100.0**	0.0	0.0	
	strong-100		0.0	0.0	**100.0**	0.0	0.0	
**15tax-sim**	100gt-100bp	50.0	**100.0**	**100.0**	**100.0**	0.0	60.0	0.0
	100gt-1000bp	**100.0**	**100.0**	**100.0**	**100.0**	0.0	0.0	0.0
	100gt-true	0.0	0.0	0.0	**100.0**	0.0		
	1000gt-100bp	80.0	**100.0**	**100.0**	**100.0**	0.0	90.0	0.0
	1000gt-1000bp	**100.0**	**100.0**	**100.0**	**100.0**	0.0	0.0	0.0
	1000gt-true	0.0	0.0	0.0	**100.0**	0.0		
**37tax-sim**	0.5X-200-500	**100.0**	**100.0**	**100.0**	**100.0**	0.0	0.0	0.0
	1X-200-1000	**100.0**	**100.0**	**100.0**	**100.0**	0.0	0.0	0.0
	1X-200-500	**100.0**	**100.0**	**100.0**	**100.0**	0.0	0.0	0.0
	1X-400-1000	**100.0**	**100.0**	**100.0**	**100.0**	0.0	0.0	0.0
	1X-400-500	**100.0**	**100.0**	**100.0**	**100.0**	0.0	0.0	0.0
	1X-800-1000	**100.0**	**100.0**	**100.0**	**100.0**	0.0	0.0	0.0
	1X-800-500	**100.0**	**100.0**	**100.0**	**100.0**	0.0	0.0	0.0
	2X-200-500	**100.0**	**100.0**	**100.0**	**100.0**	0.0	0.0	0.0
**48tax-sim**	0.5X-1000-500	0.0	0.0	0.0	**100.0**	0.0	0.0	0.0
	1X-100-500	0.0	0.0	0.0	**100.0**	0.0	0.0	0.0
	1X-1000-500	0.0	0.0	0.0	**100.0**	0.0	0.0	0.0
	1X-200-500	0.0	0.0	0.0	**100.0**	0.0	0.0	0.0
	1X-25-500	0.0	0.0	0.0	**100.0**	0.0	0.0	0.0
	1X-50-500	0.0	0.0	0.0	**100.0**	0.0	0.0	0.0
	1X-500-500	0.0	0.0	0.0	**100.0**	0.0	0.0	0.0
	2X-1000-500	0.0	0.0	0.0	**100.0**	0.0	0.0	0.0
**200tax-sim**	estimated	30.0	40.0	40.0	**100.0**	0.0		
**500tax-sim**	true	40.0	0.0	0.0	**100.0**	0.0		
	estimated	10.0	20.0	30.0	**100.0**	0.0		

**Table 9:** To gain better insights into STELAR’s ability to correctly infer species tree roots, we calculate the Average Topological Distance between true and estimated roots of species trees inferred from the sets of rerooted gene trees. Bold values indicate the best entry in a row.

Dataset	Model Cond^*n*^	MAD	MP	MV	OG	RAND	RD	RDEX
**11tax-sim**	strong-5		1.0	1.0	**0.0**	2.0		
	strong-15		1.0	1.0	**0.0**	1.2		
	strong-25		1.0	1.0	**0.0**	1.1		
	strong-50		1.0	1.0	**0.0**	1.0		
	strong-100		1.0	1.0	**0.0**	1.0		
**15tax-sim**	100gt-100bp	1.1	**0.0**	**0.0**	**0.0**	2.8	0.4	3.6
	100gt-1000bp	**0.0**	**0.0**	**0.0**	**0.0**	4.4	2.5	4.0
	100gt-true		4.9	5.6	**0.0**	6.0		
	1000gt-100bp	0.2	**0.0**	**0.0**	**0.0**	3.0	0.1	3.3
	1000gt-1000bp	**0.0**	**0.0**	**0.0**	**0.0**	5.5	3.0	4.8
	1000gt-true		5.0	5.9	**0.0**	5.8		
**37tax-sim**	0.5X-200-500	**0.0**	**0.0**	**0.0**	**0.0**	4.1	4.5	2.9
	1X-200-1000	**0.0**	**0.0**	**0.0**	**0.0**	4.1	4.1	3.1
	1X-200-500	**0.0**	**0.0**	**0.0**	**0.0**	4.1	4.2	3.0
	1X-400-1000	**0.0**	**0.0**	**0.0**	**0.0**	4.0	4.1	3.0
	1X-400-500	**0.0**	**0.0**	**0.0**	**0.0**	4.1	4.1	3.0
	1X-800-1000	**0.0**	**0.0**	**0.0**	**0.0**	4.0	4.0	3.0
	1X-800-500	**0.0**	**0.0**	**0.0**	**0.0**	4.0	4.0	3.0
	2X-200-500	**0.0**	**0.0**	**0.0**	**0.0**	4.0	4.1	2.9
**48tax-sim**	0.5X-1000-500	2.0	2.0	2.0	**0.0**	4.7	8.6	2.0
	1X-100-500	5.7	2.8	4.0	**0.0**	6.0	8.0	3.4
	1X-1000-500	4.1	2.0	3.2	**0.0**	5.0	8.6	3.0
	1X-200-500	4.7	2.4	3.8	**0.0**	5.5	8.4	3.2
	1X-25-500	6.2	2.7	4.6	**0.0**	6.8	7.0	4.6
	1X-50-500	6.1	2.6	4.0	**0.0**	6.0	7.3	4.3
	1X-500-500	4.3	2.0	3.4	**0.0**	4.8	8.4	3.0
	2X-1000-500	5.0	2.1	3.9	**0.0**	4.7	8.9	3.4
**200tax-sim**	estimated	1.0	1.0	1.1	**0.0**	3.5		
**500tax-sim**	true	3.1	2.2	2.5	**0.0**	6.4		
	estimated	3.4	4.9	3.4	**0.0**	7.0		

#### 4.1.2 15tax-sim.

A general trend similar to that in the 37tax-sim dataset is also observed in the analysis of the 15tax-sim dataset: all methods, including STELAR with different gene tree rootings, yield better performances with “easier” model conditions. However, a primary point of interest in this dataset is the significantly better performance of STELAR-MAD in terms of RF scores (as seen in Fig 3), even outperforming ASTRAL in all model conditions (except for the cases of 100gt-true and 1000gt-true, since the branch lengths were not present in these cases and the MAD rooting method cannot be run on such gene trees). This phenomenon was a more prominent variation of RD-EX’s performance in the 37tax-sim dataset. Despite displaying lower triplet scores compared to the other STELAR variants (Table 4), and lower quartet scores compared to ASTRAL (Table 5), STELAR-MAD showed better robustness to gene tree estimation errors as reflected in its mostly unmatched true triplet scores (Table 10) and true quartet scores (Table 11). Subsequent discussions on this dataset focus more on a comparative analysis involving methods other than the evidently best-performing STELAR-MAD.

**Fig 3:**
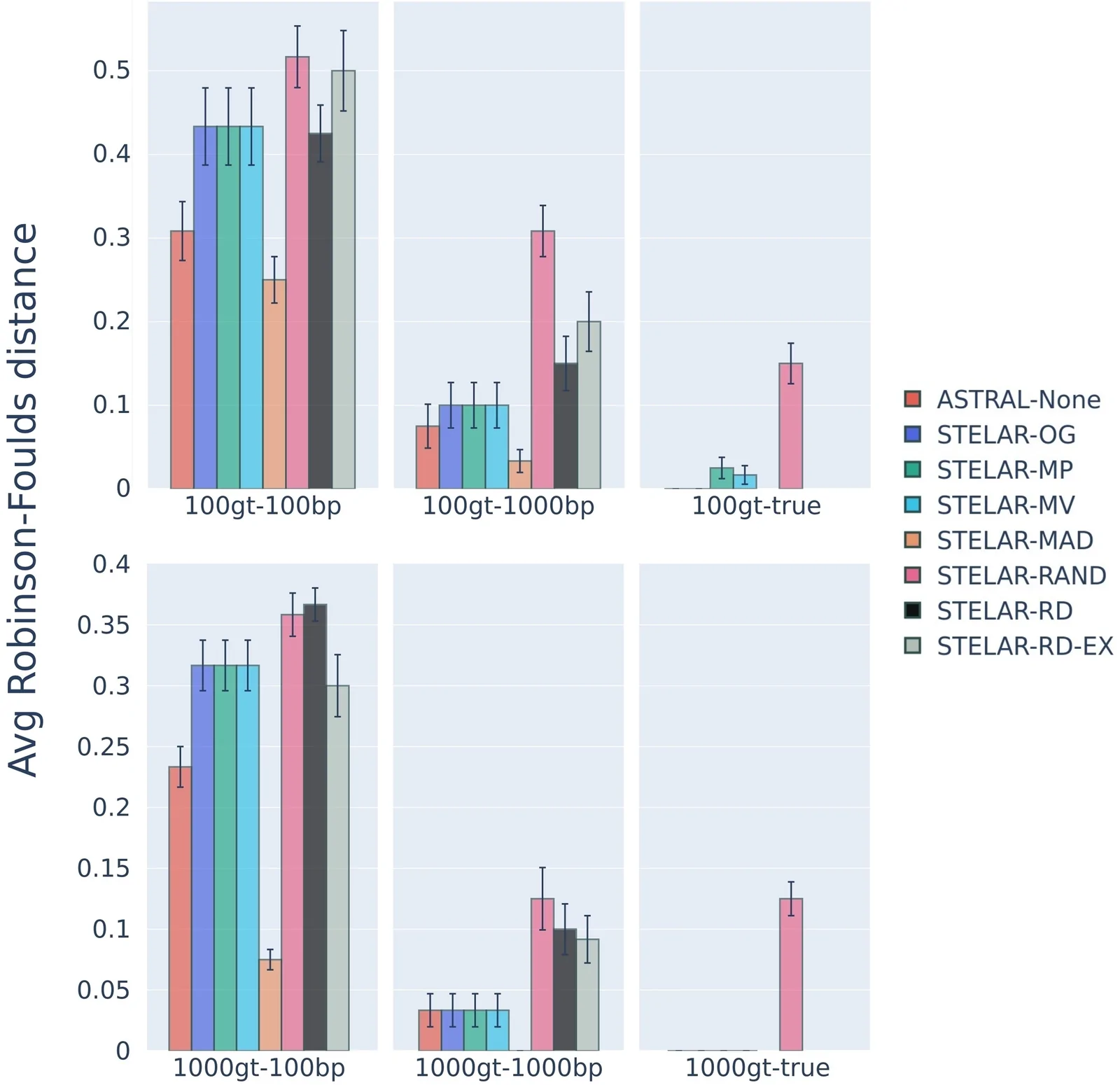
Comparison of Robinson-Foulds distance for ASTRAL and STELAR with different rooting methods on the 15tax-sim dataset. We varied the number of estimated gene trees (100genes - 1000genes) as well as the sequence length (100 bp - 1000 bp). We also analyzed model conditions with true gene trees; however, due to absence of branch lengths, we could not run MAD on these cases (100-true and 1000-true).

**Table 10:** We report the Average True Triplet Scores (TTS) of the species trees inferred by STELAR on gene trees rooted by different techniques (e.g., MP, MV, etc.). We boldfaced and normalized the values similar to Table 4.

Dataset	Model Cond^*n*^	MAD	MP	MV	OG	RAND	RD	RDEX
**11tax-sim**	strong-5		.8005	.8005	**.9459**	.6769		
	strong-15		.8262	.8262	**.9727**	.7823		
	strong-25		.8303	.8303	**.9758**	.8073		
	strong-50		.8314	.8314	**.9758**	.8179		
	strong-100		.8319	.8319	**.9768**	.8244		
**15tax-sim**	100gt-100bp	.6600	**.6695**	**.6695**	**.6695**	.4430	.6203	.4719
	100gt-1000bp	**.7202**	.7148	.7148	.7148	.4586	.5786	.5238
	1000gt-100bp	**.7055**	.6795	.6795	.6795	.4353	.6368	.4789
	1000gt-1000bp	**.7201**	.7183	.7183	.7183	.4378	.5777	.5097
**37tax-sim**	.5X-200-500	.8031	.8031	**.8033**	.8032	.5925	.5923	.7398
	1X-200-1000	.9076	**.9078**	**.9078**	.9078	.6427	.5360	.8246
	1X-200-500	.9076	**.9076**	.9076	.9070	.6423	.5596	.8313
	1X-400-1000	**.9075**	.9075	.9075	.9075	.6427	.6426	.8247
	1X-400-500	.9074	**.9074**	**.9074**	.9074	.6424	.6422	.8246
	1X-800-1000	**.8815**	**.8815**	**.8815**	**.8815**	.6244	.5064	.8010
	1X-800-500	**.8815**	**.8815**	**.8815**	**.8815**	.6244	.5423	.8009
	2X-200-500	.9636	.9636	**.9646**	.9630	.6659	.6656	.8735
**48tax-sim**	.5X-1000-500	.5811	.6415	.5862	**.6667**	.4792	.4524	.6487
	1X-100-500	.4237	.6518	.5309	**.7234**	.5192	.5071	.6399
	1X-1000-500	.4533	.6976	.5267	**.7280**	.5187	.4983	.6824
	1X-200-500	.4449	.6761	.5238	**.7247**	.5213	.5020	.6725
	1X-50-500	.4304	.6607	.5432	**.7219**	.5202	.5104	.6285
	1X-500-500	.4521	.6979	.5235	**.7263**	.5258	.4994	.6787
	2X-1000-500	.4171	.7224	.4832	**.7725**	.5517	.5253	.6807

**Table 11:** We calculate the Average True Quartet Scores (TQS) of the estimated species trees inferred by ASTRAL and STELAR paired with different rooting techniques, with values boldfaced and normalized similar to Table 5.

Dataset	Model Cond^*n*^	ASTRAL	STELAR						
			MAD	MP	MV	OG	RAND	RD	RDEX
**11tax-sim**	strong-5	**.8436**		.8406	.8406	.8409	.8312		
	strong-15	.9118		.9126	.9126	**.9159**	.8634		
	strong-25	.9239		**.9243**	**.9243**	**.9243**	.8828		
	strong-50	.9287		**.9290**	**.9290**	.9262	.8936		
	strong-100	**.9306**		**.9306**	**.9306**	.9292	.9103		
**15tax-sim**	100gt-100bp	.6019	**.6082**	.5899	.5899	.5899	.5744	.5905	.5783
	100gt-1000bp	.6179	**.6194**	.6163	.6163	.6163	.6051	.6136	.6108
	1000gt-100bp	.6066	**.6170**	.5985	.5985	.5985	.5972	.5922	.6030
	1000gt-1000bp	.6179	**.6185**	.6179	.6179	.6179	.6132	.6152	.6134
**37tax-sim**	.5X-200-500	.7807	**.7807**	.7806	.7807	.7807	.7801	.7795	.7806
	1X-200-1000	.8891	.8891	**.8892**	**.8892**	.8892	.8887	.8888	.8890
	1X-200-500	.8888	.8888	**.8889**	.8888	.8888	.8884	.8880	.8887
	1X-400-1000	.8890	**.8891**	.8890	.8890	.8890	.8885	.8883	.8890
	1X-400-500	**.8889**	.8888	.8889	.8889	.8888	.8883	.8875	.8888
	1X-800-1000	**.8637**	**.8637**	**.8637**	**.8637**	**.8637**	.8634	.8632	**.8637**
	1X-800-500	**.8636**	**.8636**	**.8636**	**.8636**	**.8636**	.8633	.8630	.8636
	2X-200-500	.9513	.9509	.9514	**.9515**	.9513	.9508	.9499	.9515
**48tax-sim**	.5X-1000-500	**.6142**	.5681	.6051	.5704	.6123	.6071	.6083	.6117
	1X-100-500	**.6973**	.6707	.6809	.6658	.6915	.6866	.6843	.6856
	1X-1000-500	**.6989**	.6672	.6882	.6414	.6977	.6903	.6920	.6901
	1X-200-500	**.6982**	.6675	.6852	.6644	.6939	.6878	.6860	.6873
	1X-50-500	**.6965**	.6732	.6749	.6663	.6896	.6854	.6795	.6839
	1X-500-500	**.6978**	.6672	.6867	.6529	.6957	.6887	.6905	.6895
	2X-1000-500	**.7626**	.7357	.7434	.7156	.7616	.7462	.7524	.7467

STELAR exhibits the same robustness to gene tree rooting in this dataset as seen in the 37tax-sim dataset. In terms of species tree accuracy, comparing the relative performance of STELAR with different rooting techniques shows that STELAR-MP and STELAR-MV performed on par with STELAR-OG, except in the 100gt-true case where STELAR-OG performed better than the MP and MV variants (though not statistically significant). Although STELAR-RD performed better than the other rooting methods on the “unfavorable” model conditions with small numbers of genes and short sequence lengths (100gt - 100 bp), it gradually fell off with easier model conditions. A somewhat opposite trend can be observed for STELAR-RD-EX, where it performed worse in 100gt cases, but better in the 1000gt-100 bp model condition. STELAR with random rooting yielded competitive performances in some cases (100 bp), despite worse results in most other conditions. This drop in performance for STELAR-RAND was more significant than that in the 37tax-sim dataset, indicating a possible correlation between its worse performance and the lower number of taxa.

To reconfirm generalization beyond STELAR, we also conducted experiments with SuperTriplets on the 15tax-sim dataset. While we observed an improved performance of the RootDigger variants (RD and RD-EX) under “difficult” conditions, e.g., 100 bp (detailed in Section 3.2 of S1 Appendix), the overarching RF score trends remained qualitatively similar (Fig B of S1 Appendix).

A comparison of the performance of ASTRAL with the STELAR variants on this dataset shows that, as previously stated, STELAR-MAD yields significantly better performances in all model conditions. STELAR-RD could not see an improvement from its performance in the previous dataset. Rather, STELAR-RD-EX failed to replicate its success from our prior analysis. Considering the rooting methods MP, and MV, STELAR yielded the same species trees as ASTRAL under conditions of 1000gt-1000 bp and 1000gt-true, and showed competitive performances in 100gt-1000 bp and 100gt-true. However, unlike similar conditions in the 37tax-sim, lower basepairs corresponded to worse performances in general by STELAR; with notable differences from ASTRAL in 100gt-100 bp and 1000gt-100 bp.

When trying to correlate the triplet scores to performances in terms of RF scores, we notice a trend different from that observed in the 37tax-sim dataset. STELAR-MAD yielded the lowest triplet scores in most cases (Table 4). However, we see that triplet scores are not the best indicator of species tree accuracy in this dataset, since STELAR-MAD had the best performance in terms of RF scores. STELAR-MP, STELAR-MV and STELAR-OG yielded the same triplet score in all cases except the true-gt cases where STELAR-MP came in second after STELAR-OG, followed by STELAR-MV in last place. When we look at the quartet scores for each estimated tree, we see ASTRAL had the highest quartet scores in all cases (a performance akin to the results in the 37tax-sim dataset), whereas STELAR-MAD yielded the best quartet scores among the STELAR variants (Table 5). Comparing the higher quartet scores of STELAR-MAD with its RF-scores shows that quartet scores were better correlated to species tree accuracy in this dataset.

Analysis of the percentage of correctly rooted gene trees (Table 6), and the average topological distance between true root and inferred roots (Table 7) yields fascinating insights into the relationship between gene tree rooting and the robustness of STELAR to gene tree estimation error, which were not evident from our analysis of the 37tax-sim dataset results. All rooting methods failed to correctly root the gene trees in the true-gt cases, and the RD variants both yielded poor performances in most cases in terms of these metrics. Though MP and MV correctly predicted the gene tree rootings in all other cases, MAD actually rooted the gene trees at points other than the outgroup quite frequently (getting the “correct” root around 20% and 80% of the time in the 100 bp and 1000 bp cases respectively, from Table 6). A similar trend is reflected in the average topological distances in Table 7 as well. However, this seemingly “inaccurate” rooting by MAD actually helped STELAR in addressing gene tree estimation errors to an extent, resulting in the best performances in this dataset as evident from prior discussions.

Looking at the corresponding metrics for species tree rooting accuracy from Tables 8 and 9 reveals that STELAR, unsurprisingly, always predicted the correct species tree rooting when the gene trees were correctly rooted (e.g., STELAR-MP and STELAR-MV in cases other than true-gt). However, interestingly, despite the inaccuracies in gene tree rooting by MAD in the 1000 bp cases, STELAR always correctly predicted the species tree roots. Furthermore, STELAR-MAD achieved accuracies of 50% and 80% in species tree root prediction despite having around 20% accuracy in gene tree rooting in the 100gt-100 bp and 1000gt-100 bp cases respectively. Moreover, the average topological distances between the predicted species tree root and the true root were only around 1 and 0.2 in these cases, respectively. This attests to the robustness of STELAR to gene tree rooting in the case of species tree root prediction, as also seen from prior discussions on the 37tax-sim dataset.

#### 4.1.3 11tax-sim.

With varying gene counts, the RF scores (Fig 4) depict a general trend similar to that of the 37tax-sim and 15tax-sim datasets: increasing genecounts always results in more accurate species trees, leading to lower RF scores for ASTRAL and all STELAR variants. All variants of STELAR (except STELAR-RAND) yielded almost identical performances in terms of RF scores when compared to ASTRAL, with STELAR-MP and STELAR-MV yielding the same species trees in all cases.

**Fig 4:**
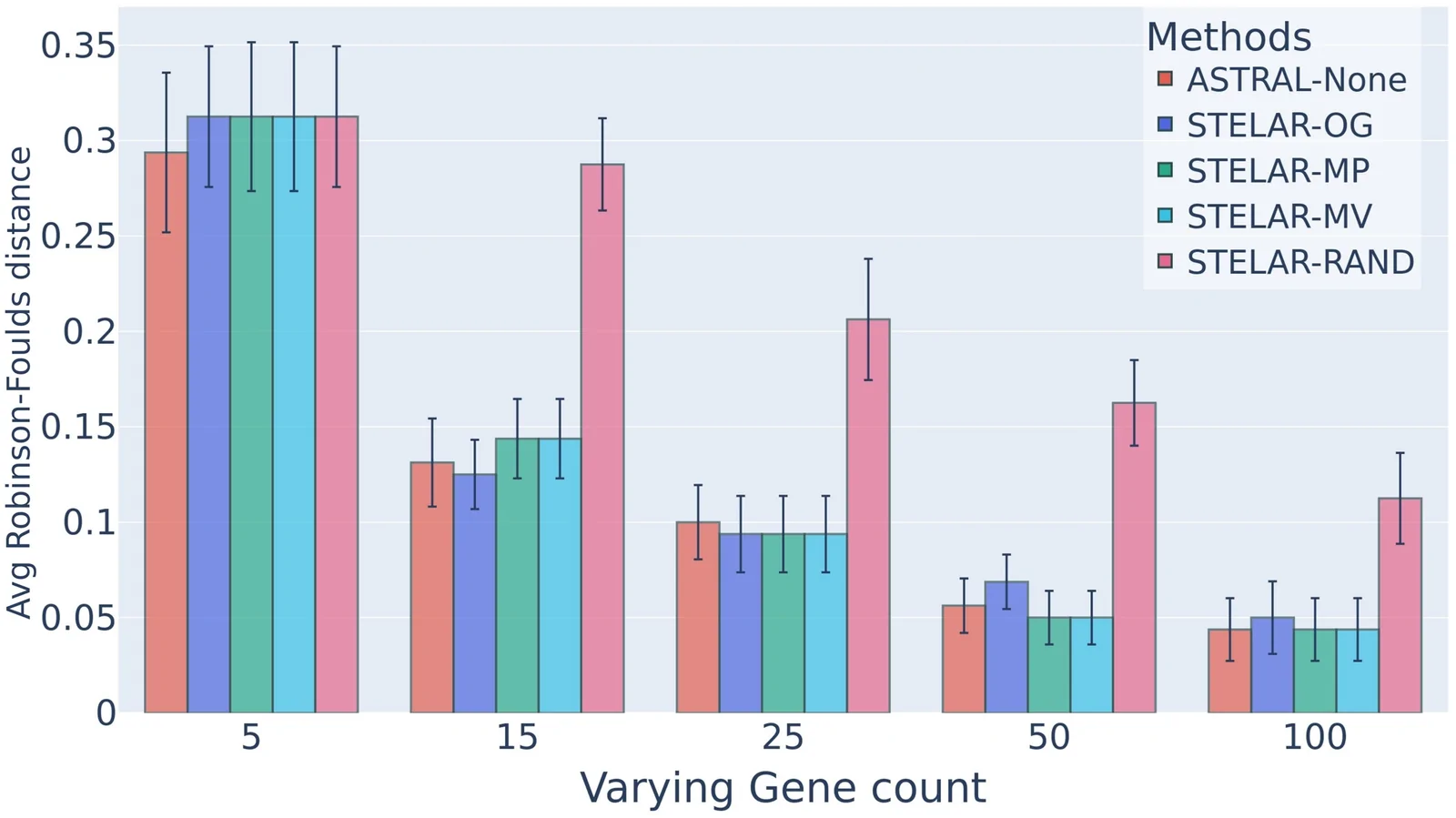
Comparison of the Robinson-Fould’s distance for ASTRAL and the STELAR variants on the 11tax-sim dataset. We varied the gene counts from 5 to 100, considering only the high ILS condition.

Compared to the previous datasets, the differences in RF scores were more subtle in this case. These could not be captured completely by the triplet scores, for example - despite having a higher triplet score than STELAR-MP and MV, in the 50 and 100 gene conditions (Table 4), STELAR-OG had a slightly worse RF score (Fig 4). Upon inspecting the True Triplet Scores (TTS) from Table 10, we find that the RF score trends were not sufficiently explained by TTS as well. Referring to the quartet scores in Table 5, we see that ASTRAL has marginally higher quartet scores in each case despite not always being the best in terms of RF scores, a trend that carries over from prior datasets. However, unlike the TTS, we find that True Quartet Scores (TQS) from Table 11 better capture the subtle changes in RF scores. We also analyzed results with SuperTriplets as the triplet-based estimation method, with details in Section 3.3 of S1 Appendix.

Referring to Table 7, we see that MV displayed a slightly higher inaccuracy in rooting the gene trees, when compared to MP. Despite this, STELAR still maintains its robustness in estimating the rooting of the final species tree in both cases (Table 9), with the estimated root differing from the true root by one node on average.

#### 4.1.4 48tax-sim.

In line with our prior observations, all methods followed the trend of better RF scores with “easier” model conditions (Fig 5). Unlike thus far, STELAR yielded better results with outgroup rooting in this particular dataset. Despite yielding competitive performances in “harder” conditions, STELAR-MP, STELAR-MV, STELAR-MAD failed to keep pace with STELAR-OG in higher gene counts (Fig 5a) and lower ILS (Fig 5b). Furthermore, STELAR proved to be more robust to random rooting - yielding the best performance by STELAR-RAND so far. This reinforces the hypothesis that RAND rooting performs better as taxa count increases. We also see that both STELAR-RD and STELAR-RD-EX yielded rather comparable results in most model conditions. As with previous datasets, we also analyzed the performance of SuperTriplets, discussed in detail in Section 3.4 of S1 Appendix.

**Fig 5:**
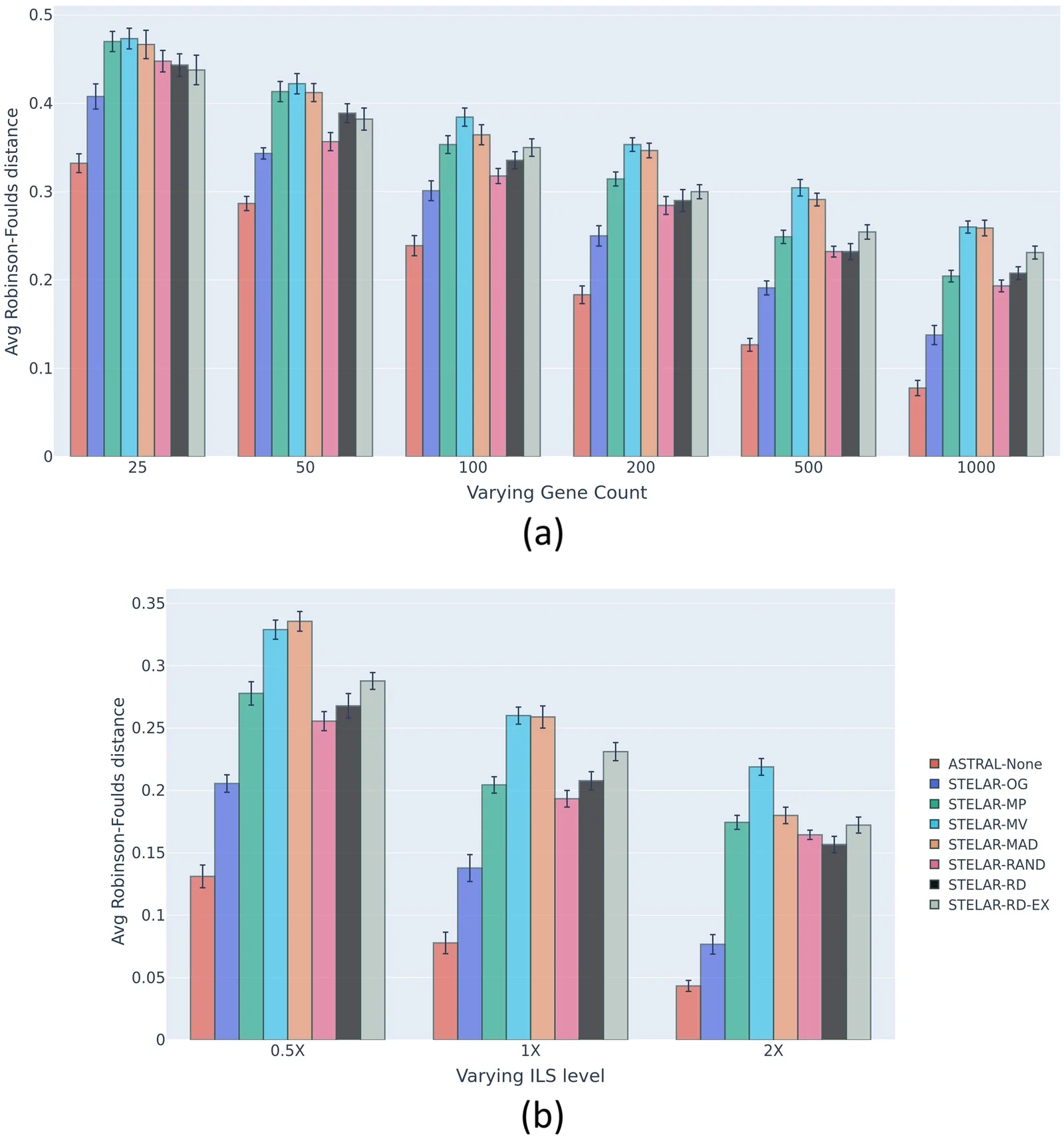
Comparison of Average Robinson-Foulds distance for ASTRAL and STELAR with different rooting methods for the 48tax-sim avian dataset. **(a)** We fixed the ILS at moderate (1X) and the base pairs at 500 bp, and varied gene counts between from 25 to 1000. **(b)** We set the gene trees at 1000, the sequence length at 500 bp, and varied the ILS between high (0.5X), moderate (1X) and low (2X).

As with previous observations, the triplet scores followed the trend of increasing with lower RF scores and vice versa (Table 4). However, exceptions were observed in this dataset for STELAR-RAND and STELAR-RD. These exceptions persisted even when looking at the True Triplet Scores (Table 10), similar to observations in the 37tax-sim dataset. On the other hand, quartet scores (Table 5) were a better indicator of RF-scores in this dataset - modeling the trend that “higher quartet scores mean lower RF scores” better than previous datasets. The few discrepancies in this trend are all solved if we look at the True Quartet Scores (TQS) from Table 11, where a higher TQS almost always means a lower RF score, which was not always the case in the 37tax-sim dataset.

In order to explain the cause for the decline in performance for STELAR-MP, STELAR-MV and STELAR-MAD, we inspect the quality of gene tree rooting depicted in Table 7. Compared to the previous datasets, we find gross inaccuracies in gene tree rooting for MV and MAD, as well as for MP in a slightly less severe sense. Since the rooting methods MP, MV, and MAD are closely dependent on the branch lengths and branch supports of the input gene trees, we suspect irregularities in these values for this 48tax-sim dataset, leading to worse gene tree rootings. This irregularity in gene tree rooting led to worse performance for the corresponding STELAR variants. Similar to 15tax-sim and 37tax-sim datasets, gene tree rootings by RD and RAND had very high topological distances from the true root, leading to lower TS and TTS. However, unlike before, their RF scores were indeed competitive this time, as discussed above. Furthermore, unlike before, STELAR had a difficult time predicting the root this time, as indicated by none of the variants correctly predicting the root (Table 8) and the high topological distances between species tree root and true root (Table 7).

#### 4.1.5 Results on higher number of taxa.

On the 200tax-sim dataset, STELAR-OG outperformed all other variants of STELAR in terms of RF scores by a significant margin, as seen from Fig 6a. Strikingly, RAND showed a performance comparable to that of MP, MV, and MAD. This also translated to the triplet scores, where RAND yielded the highest triplet score among all methods.

**Fig 6:**
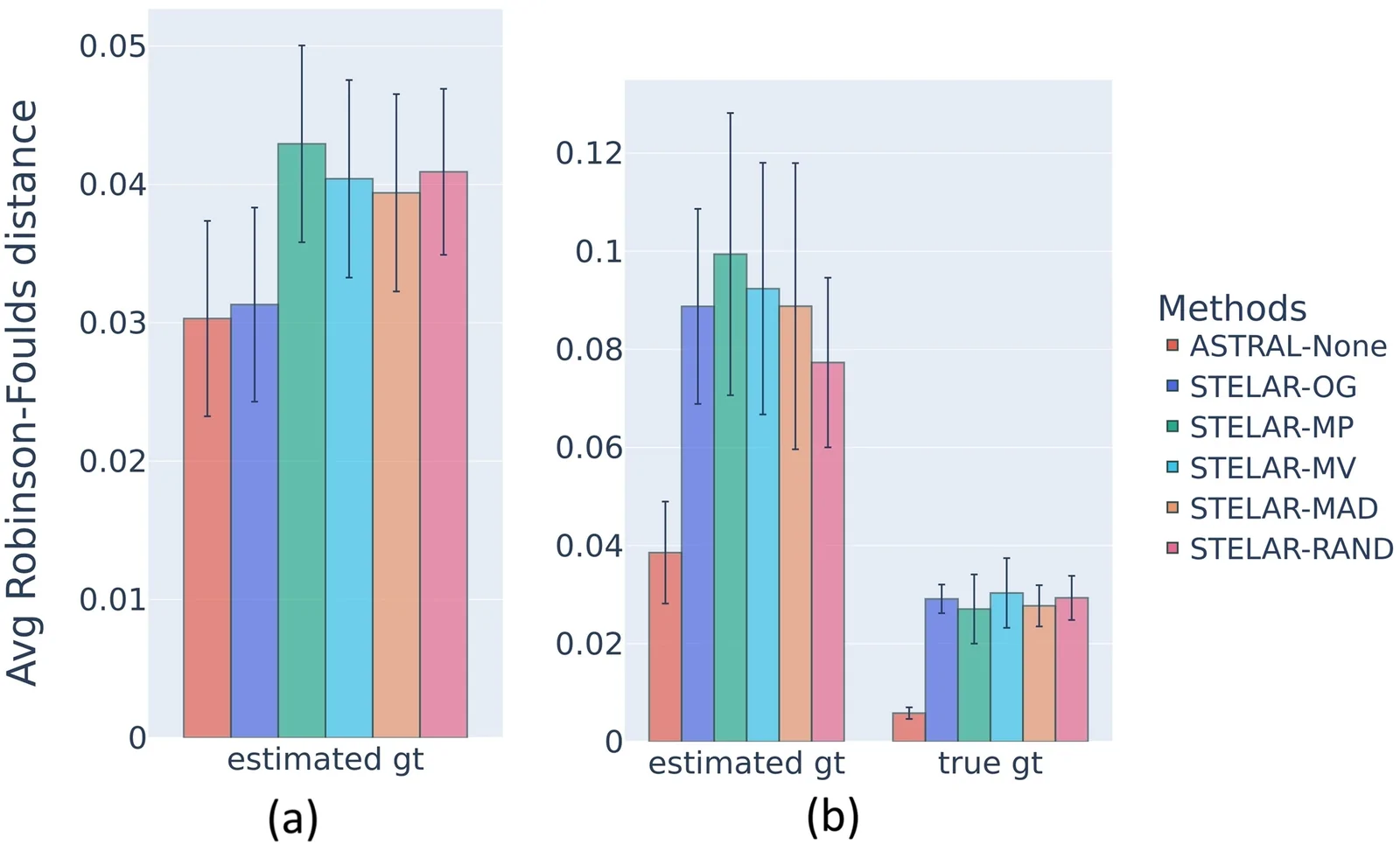
Comparison of average Robinson-Foulds distance for ASTRAL and STELAR with different rooting methods for higher-order taxa datasets. **(a)** Comparison of the RF distance for 200tax-sim dataset **(b)** Comparison of the RF distance for 500tax-sim dataset, with estimated gene trees and true gene trees respectively.

A similar robustness to gene tree rooting for STELAR is noticed in the 500tax-sim dataset depicted in Fig 6b. In the simulated case, STELAR-RAND came in second, outperforming even STELAR-OG in terms of RF scores. STELAR-RAND also showed a performance comparable to the other STELAR variants in the true gene tree case. However, ASTRAL displayed statistically significant improvement in terms of RF scores when compared to the STELAR variants. We also look at the performance of SuperTriplets, and interestingly, it yields better performances in general compared to STELAR on the 500tax-sim experiments (details in Section 3.5 of S1 Appendix.)

The number of species trees in the search space that differs from the “best” tree by *k* edges increases as the number of taxa increases. As such, STELAR has more options to arrive at a species tree with a better/same RF score, despite working on randomly rooted gene trees. Furthermore, a single mismatched bipartition has a more severe impact on the RF score when the number of taxa is lower. The better performance of STELAR-RAND in terms of RF score for large datasets can possibly be attributed to these facts. Thus, STELAR grows increasingly robust to gene tree rooting as the number of taxa increases.

### 4.2 Biological datasets

#### 4.2.1 Amniota dataset.

We analyzed the nucleotide (DNA) variant of the dataset from [80], containing 16 Amniota taxa. A key challenge lies in resolving the position of turtles relative to crocodiles and birds. Past studies like [13] place turtles as sisters to the archosaurs clade (comprised of birds and crocodiles). Furthermore, [83] analyzes the Squamates clade in detail, and helps identify the Toxicofera clade in our study.

The species trees recovered by all the methods, rerooted at *Protopterus* for ease of visualization, are depicted in Fig 7. We also summarize clade recovery across rooting methods using the grid-based presentation shown in Table 2, inspired by [84]. ASTRAL and the STELAR variants OG, and unexpectedly, RAND predicted all the significant clades correctly (Fig 7a). STELAR-MV could not recover the Toxicofera clade (Fig 7b) and in addition, STELAR-MAD and STELAR-MP also failed to recover the Archosaurs clade (Fig 7c). STELAR-RD misplaced Xenopus, thus being the only method that failed to recover the monophyly of the Amniota clade (Fig 7d). And STELAR-RD-EX misplaced the Alligator and Caiman taxa, errors that have significant effects on the topology of the inferred species trees (Fig 7e). Similar to simulated datasets, the quartet scores (Table 5) more closely predicted the relative performance of the methods compared to the triplet scores (Table 4).

**Fig 7:**
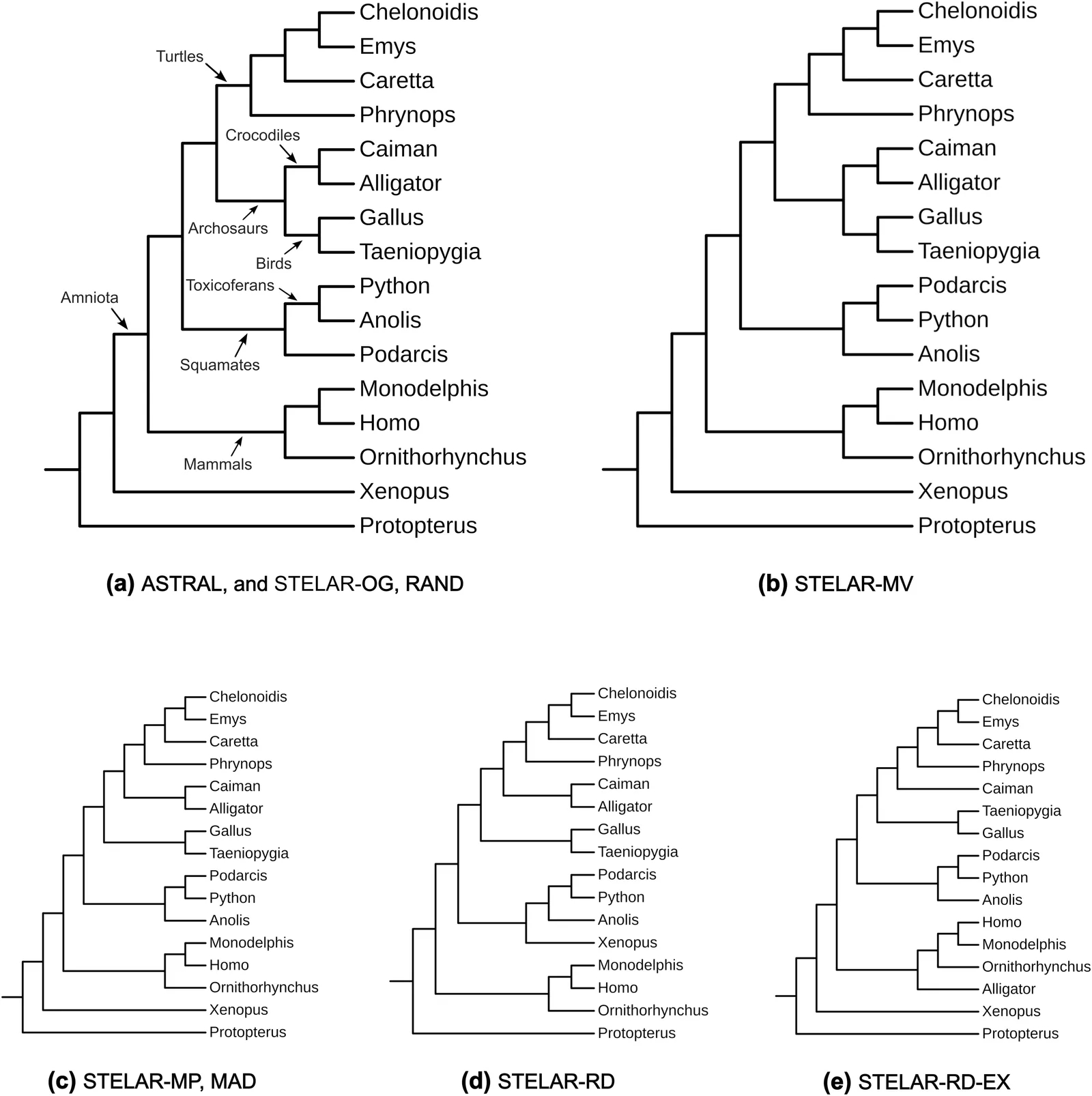
Species trees inferred by different methods on the Amniota nucleotide biological dataset. All trees are rooted using *Protopterus* for ease of comparison. **(a)** Our reference species tree, reported in [13], which is the same as the tree inferred by ASTRAL, STELAR-OG, and STELAR-RAND. We label the following noteworthy clades: Turtles, Crocodiles, Birds, Archosaurs, Toxicoferans, Squamates, Mammals and the Amniota ingroup. **(b)** The species tree inferred by STELAR-MV; the clade Toxicofera could not be recovered. **(c)** STELAR-MP and STELAR-MAD could not recover the Archosaurs and Toxicofera clades. **(d)** STELAR-RD, in addition, also misplaced Xenopus in the Amniota ingroup. **(e)** STELAR-RD-EX could predict the Turtles, Birds, Squamates, Mammals and the Amniota ingroup clades correctly.

For this dataset, the expected biological root is *Protopterus* as the outgroup [13,80]. Among the methods evaluated, the STELAR variants OG, MAD, MV, and RD inferred a root consistent with this expectation. STELAR-MP instead placed the root on the branch separating *(Protopterus, Xenopus)* from the remaining taxa. In contrast, STELAR-RD-EX inferred a substantially different root, splitting the mammalian clade *((Ornithorhynchus, (Homo, Monodelphis)))* from all other taxa, representing the most discordant root placement among the methods considered.

#### 4.2.2 Angiosperm dataset.

Analyses on this dataset aim to address long-standing questions in the phylogeny of Angiosperms, with the key challenges lying in the phylogenetic placement of *Amborella trichopoda* and its relative branching order with other lineages like Nymphaeales (i.e., *Nuphar* or waterlilies).

STELAR-MAD correctly placed *Amborella* as sister to the other angiosperms and the clade of all angiosperms as sister to the outgroup (Selaginella, (Zamia, (Pinus, Picea))) (Fig 8b). But, this method could not predict *Nuphar* as the sister to the angiosperms after *Amborella* (as inferred in [77]), nor does it predict the clade of (Amborella, Nuphar) as done in [22]. The other STELAR variants failed to infer these relationships as well. We summarize these results using the same grid-based presentation (Table 3), adapted here to reflect key basal angiosperm relationships rather than named clade monophyly - as this is the main focus of analyses on this dataset.

**Fig 8:**
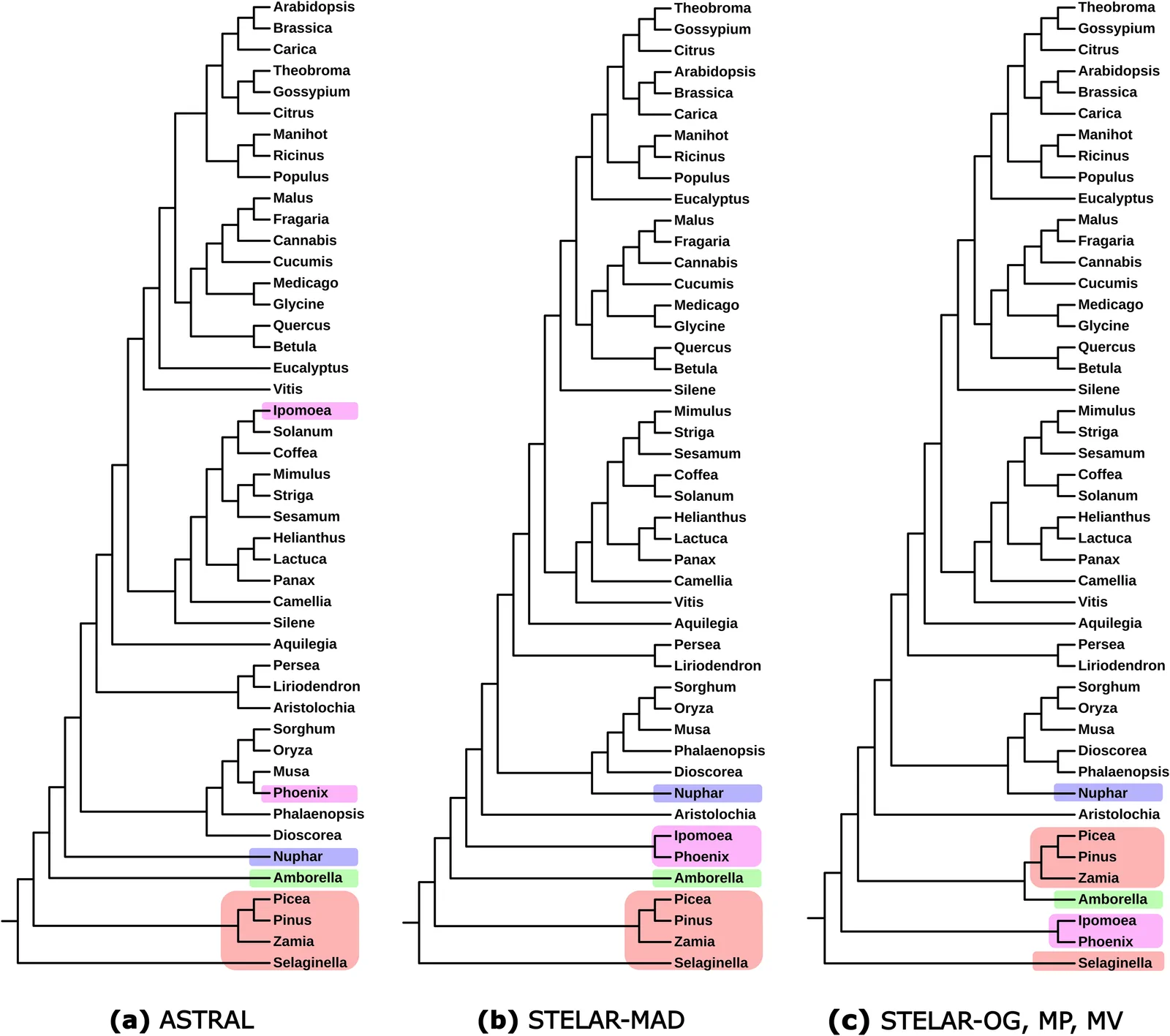
Estimated species trees for Angiosperm dataset (important clades and taxa are highlighted, and trees rerooted at an outgroup, *Selaginella*, for better visualization). Unlike the other STELAR variants, STELAR-MAD successfully predicted the placement of some important taxa/clades, yielding a species tree closer to the widely accepted one by ASTRAL. All STELAR variants predicted the root at the (incorrectly inferred) clade (*Ipomoea*, *Phoenix*).

Furthermore, unlike STELAR-MAD, the OG, MP, and MV variants of STELAR misplaced *Selaginella*, causing *Amborella* to be placed as a sister to the clade comprised of the 3 other outgroup taxa (Zamia, (Pinus, Picea)) (Fig 8c). All STELAR variants had issues with the following placements: swapped positions of *Vitis* and *Silene*, misplaced *Eucalyptus*, *Phoenix*, *Ipomoea* and *Aristolochia*. The species tree from ASTRAL is the same as stated in [79], and does not misplace the aforementioned taxa (Fig 8a). It predicts *Nuphar* as sister to the other angiosperms after *Amborella*.

*Selaginella* was the outgroup in this dataset [79]. However, it is worth noting that none of the methods originally rooted the estimated species trees at the outgroup. All the STELAR variants predicted the root at *(Phoenix, Ipomoea)*. For ease of discussion, the species trees were all rerooted at *Selaginella*.

#### 4.2.3 Mammalian dataset.

We analyzed the Mammalian Dataset from [71], containing 37 taxa and 424 genes. Our aim was to study the impact of different rooting methodologies applied to the input gene trees on the species tree inferred by STELAR.

We found that ASTRAL and all the variants of STELAR inferred the same species tree (Fig C of S1 Appendix). Our inferred mammalian phylogeny is also broadly consistent with recent large-scale phylogenomic studies, including the comprehensive genomic analysis of 241 placental mammals by [85].

In this dataset, the biologically supported root separates *Gallus* from all mammalian taxa. STELAR-MV, STELAR-MAD, and STELAR-OG recovered the root at *Gallus* whereas STELAR-MP and STELAR-RD inferred the root at the clade (Gallus, Ornithorhynchus) in the species tree. STELAR-RD-EX inferred the species tree root at *(Macropus, Monodelphis)*

In conclusion, STELAR demonstrated robustness in inferring species trees regardless of the rooting techniques applied. Even when random rooting was used on the input gene trees, STELAR was able to infer the same species tree. This indicates that, for the Mammalian dataset, the species tree inference by STELAR was unaffected by the rooting of the input gene trees.

### 4.3 Summary of metrics

In this subsection, we present consolidated summaries of the evaluation metrics across all simulated and empirical datasets.

## 5 Discussion

Both quartets and triplets avoid the “anomaly zone” – a scenario where the most likely gene tree topology may differ from the species tree topology [18–20]. Thus, statistically consistent methods have been developed by maximizing quartet and triplet consistency. While quartet-based summary methods like ASTRAL are in wide use, triplet-based methods like STELAR has not gained notable attention from the community. Moreover, triplet-based methods require rooted gene trees which is often difficult to obtain in the absence of molecular clock or reliable outgroups. Consequently, various techniques to root a given set of unrooted gene trees have been developed, differing in the type of data that can be analyzed, the assumptions about the evolutionary dynamics of the data, and their scalability or general applicability. However, little is known about the impact of these rooting techniques on species tree inference.

In this study, we considered a broad range of rooting approaches and their effects on species tree inference by maximizing triplet consistency. We found that STELAR was robust to the choice of rooting in most simulated model conditions as well as on real biological datasets (e.g., angiosperm dataset); where gene trees with algorithmic rootings performed competitively when compared to those rooted at the outgroup. The effect of gene tree rooting on species tree accuracy diminished with increasing taxa, to an extent where random rooting performed on par with the other methods in 200 and 500tax-sim datasets. Randomness in rooting also seemed to have less impact in “more difficult” model conditions (higher ILS, lower basepairs, lower gene counts). These observations attest to the acceptability of STELAR in datasets where the outgroup is difficult to infer.

In addition, our studies also revealed conditions that yielded notable differences in species tree accuracy based on different gene tree rooting techniques. More interestingly, under some model conditions (like 0.5X-200–500, 1X-200–500, 2X-200–500, and 1X-200–1000 for 37tax-sim and almost all cases for 15tax-sim), STELAR produced more accurate species trees with rootings other than at the known outgroup. In the cases mentioned above, STELAR with algorithmic rootings also outperformed the state-of-the-art species tree estimation methods ASTRAL in terms of accuracy, suggesting that ‘correct’ rootings can lead to significant improvements in performance for STELAR.

Furthermore, we investigated triplet and quartet scores to better understand the effect of gene tree rooting on the performance of STELAR. Both of these metrics are statistically significant, but under practical model conditions with limited numbers of genes with gene tree estimation errors, achieving the highest quartet scores (as done by ASTRAL) or the highest triplet score (STELAR-OG) does not necessarily correspond to the best RF score, as seen in many model conditions. However, throughout our experimentation, we observe that quartet scores tend to be more correlated with the RF score than triplet scores.

To estimate the error in rooting, we calculate - for each method - the percentage of “correct” roots (w.r.t the true root) both for the input gene trees and for the species trees inferred by STELAR. Our study shows that STELAR is quite adept at predicting the correct root for the output species tree, even when the input gene trees have substantially different rootings (as clearly seen in the 37tax-sim dataset). We also adopt the topological distance metric introduced by [30], using its non-normalized variant to quantify the distance between the inferred and true root(s), thereby assessing the effect of rooting error on STELAR’s performance.

One such significant insight is the ability of STELAR to address gene tree estimation error when provided appropriately rooted gene trees as input. We affirm this by calculating the triplet scores and quartet scores of STELAR outputs with respect to the true gene trees (gene trees free from all forms of estimation errors) - naming these metrics as true triplet scores and true quartet scores respectively. With increasing gene tree estimation error, maximizing the triplet score by rooting at/near the outgroup will not necessarily yield species trees with the best RF score. Certain algorithmic rooting methods can figure out roots that reduce the effect of this gene tree estimation error on STELAR’s performance. A noteworthy instance of this phenomena is the performance of STELAR-MAD in the 15tax-sim dataset, where a seemingly “uncommon” root further from the true root helped it perform significantly better than the other methods.

To test whether the robustness of STELAR carries over to real-world biological datasets, we ran experiments on three empirical datasets. We found that STELAR shows similar robustness to gene tree rooting, as it yielded similar performances with algorithmically rooted inputs when compared to gene trees rooted at the outgroup. Furthermore, STELAR when paired with algorithmic rootings succeeded in predicting some important clades (like the Archosaurs clade in the Amniota-NT dataset), which was not the case for STELAR paired with outgroup rooting.

Despite our thorough experimentation across diverse datasets and model conditions, we acknowledge limitations and potential areas for extension. Firstly, we explored datasets where the primary source of gene tree discordance was from Incomplete Lineage Sorting (ILS); this study can be extended to datasets where discordance arises from Horizontal Gene Transfer as well as gene duplication and loss. Furthermore, given the mixed performance of various rooting techniques, relative performance on finite data clearly depends on the model conditions. Hence, we do not make any general recommendation in favor of one type of rooting over another. And although we observe certain strengths for each method (e.g., low ILS conditions for MV, high ILS for RD-EX, generally good performance for MP, high gene tree estimation error for MAD), extending our experiment to diverse datasets with finer control over the dataset generation parameters (like true species tree topology, varying speciation rates, controlled rates of gene tree estimation errors) will help us further pinpoint the strengths and weaknesses of each method. Moreover, the preprocessing steps for the rooting methods can be improved: for example, MP, MV, and MAD rooting methods all require, and are heavily influenced by, branch lengths. Thus in datasets without branch lengths, we set each branch to have unit length, which sometimes adversely affected STELAR’s performance (e.g., MP, MV variants in the 48tax-sim dataset and the 100gene-true model condition of the 15tax-sim dataset). This can be improved by using branch length estimation algorithms like [86], and could be another aspect of research. In addition, our study focuses on a posteriori rooting of fixed gene tree topologies. An important extension would be to evaluate Bayesian time-tree approaches that jointly estimate topology, divergence times, and root position under birth–death or coalescent models, thereby incorporating rooting directly into the inference framework. Comparing such joint approaches with post hoc rooting strategies would clarify how model assumptions influence root placement. Future work could further evaluate asymmetry-based rooting approaches such as RD and RD-EX using simulations generated under explicitly non-reversible substitution models. Such analyses would better reflect the conditions under which these methods are intended to operate and provide a more comprehensive assessment of their performance. Moreover, while our experiments were conducted under the multispecies coalescent (ILS-driven discordance), extending similar analyses to reconciliation-based frameworks that explicitly model gene duplication and loss (GDL) would be valuable, as rooting behavior may interact differently with duplication–loss processes. Furthermore, it was not possible to note the quartet scores and triplet scores (using the functionalities provided by the ASTRAL and STELAR softwares respectively) for large datasets (e.g., 500tax-sim) due to overflow errors. In the future, integrating efficient algorithms similar to those proposed by [87] into the scoring procedures of these tools may help mitigate scalability and overflow issues, and thus represents a promising direction for future work.

## Supporting information

S1 Appendix Supplementary material includes dataset simulation procedures, results for the method Supertriplets, results on the biological mammalian dataset, supplementary tables (A-D), and figures (A-F). (PDF)
