# Supplementary material for: Triplet-based species tree estimation: Sensitivity to gene tree rooting (or lack thereof)
Source: PLoS Comput Biol. 2026 Sep 23;22(9):e1014782. doi: 10.1371/journal.pcbi.1014782 (PMC13645218; doi:10.1371/journal.pcbi.1014782)
Supplement: Supplementary file 1 [file pcbi.1014782.s001.pdf]

S1 Appendix to

Triplet-based species tree estimation: sensitivity to gene tree rooting (or lack thereof)

Tanjeem Azwad Zaman[1,$], Rabib Jahin Ibn Momin[1,$], and Md Shamsuzzoha Bayzid[1,*]

[1]Department of Computer Science and Engineering
Bangladesh University of Engineering and Technology
Dhaka, Bangladesh
[$]These authors contributed equally
[*]shams_bayzid@cse.buet.ac.bd

1 Dataset Simulation Procedure

The simulated datasets used in this study were obtained from previously published studies and were originally generated under controlled evolutionary models to evaluate phylogenetic inference methods. A brief description of the simulation procedures used to generate these datasets is provided below.

1.1 11tax-sim

The simulated 11-taxon dataset used in this study was adopted from a previously published work (1). In that study, the dataset was constructed by combining two copies of a 5-taxon asymmetric tree, with taxon 11 serving as the outgroup for both subtrees. In one copy, taxa 6 and 8 were added to examine the potential impact of increasing the number of taxa on the estimation of internal edge concordance factors (CFs). For each 11-taxon species tree topology, two branch-length configurations were considered: long internal branches (LB) and short internal branches (SB). Branch lengths were expressed in coalescent units, obtained by dividing the number of generations by the effective population size. This representation determines the expected proportions of gene trees that are concordant or discordant with the species tree under the coalescent model.

Multilocus datasets consisting of 10, 50, or 100 unlinked gene trees were simulated along the species tree, assuming an effective population size of 50,000 haploid individuals per population. The number of generations between speciation events was determined by multiplying the branch lengths in coalescent units by the effective population size.

The simulation further incorporated rate heterogeneity among lineages using HGT-simul, where a Poisson-distributed number of genomic rate-change events (mean = 3) was applied along the species tree to introduce deviations from a strict molecular clock. Lineage-specific rates were drawn from a gamma distribution with mean 1 and shape

parameter 2. Gene-specific branch length variation was additionally introduced through Poisson-distributed rate-change events along each gene tree.

DNA sequences were simulated under the Jukes–Cantor (JC) substitution model without site-specific rate variation. This simulation framework captures multiple sources of gene-level heterogeneity, including variation in evolutionary rates, departures from clock-like evolution, and gene-tree topological discordance.

Gene trees were estimated using FastTree-2 (2), RAxML (3), and *BEAST (4). For FastTree-2 (v. 2.1.3 SSE3), maximum likelihood (ML) gene trees were inferred directly from the sequence alignments using the FastTree gtr nt command. RAxML (v. 7.3.1) was run with 20 replicates per alignment (-N 20) under the GTRGAMMA model to estimate ML gene trees, and bootstrap support was calculated from 400 bootstrap replicates. Edges with less than 75% bootstrap support were contracted to produce a 75%-support version of each gene tree. *BEAST was also used to co-estimate gene trees and species trees, complementing the ML approaches.

1.2 15tax-sim

In a previous study (5), a set of 15-taxon datasets was simulated following a caterpillar-like ultrametric species tree, where two leaves, x and y were designated as siblings. All internal branches and the branches incident to x and y were set to 0.005 substitutions per site, with the remaining branch lengths determined by the ultrametricity constraint. The population size parameter ($\theta = 4N\mu$) was fixed at 0.05 across all branches, resulting in twelve short internal branches (0.1 coalescent units) in succession.

Ultrametric gene trees were generated under the Multispecies Coalescent (MSC) model using McCoal, accounting for coalescent events based on the species tree topology, branch lengths, and population size. Sequence evolution along these gene trees was simulated with bppseqgen under a GTR+γ substitution model, employing maximum likelihood (ML) for estimating sequence probabilities.

Four model conditions were considered, each with ten replicates, by varying gene lengths (100 or 1000 sites) and the number of genes (100 or 1000). This framework was used to examine the impact of gene length and the number of loci on downstream phylogenetic analyses.

1.3 37tax-sim

The simulated 37-taxon mammalian dataset was based on a previously published study (6). This dataset consisting of 37 species and 447 loci was analyzed, with an average bootstrap support of 71%. These loci were selected using stringent orthology criteria from an initial set of over 700 potential orthologs, retaining only genes for which all species were present.

To generate simulated datasets that closely resembled the empirical data, the authors designed model conditions matching the observed average bootstrap support per gene and the total number of loci. They further explored simulation scenarios by varying the number of gene trees and their bootstrap support levels to examine performance under differing gene tree reliability.

The unbinned MP-EST species tree was used as the base model, with branch lengths in coalescent units for simulations under the Multispecies Coalescent (MSC) model. To

study the effect of Incomplete Lineage Sorting (ILS), two additional model trees were generated by scaling branch lengths of the base tree: one with doubled branch lengths to reduce ILS, and one with halved lengths to increase ILS. These three model trees were then used to simulate gene trees using DendroPy following the MSC process.

Gene trees were estimated from the simulated alignments using RAxML. Branch lengths expressed in coalescent units were converted into expected substitutions per site using empirical branch length distributions from the original mammalian gene trees. Terminal branches were mapped by species, selecting lengths randomly from the corresponding empirical trees, while internal branches were assigned using a percentile-based mapping strategy to preserve realistic internal and external branch length distributions. As a result, the simulated gene trees were non-ultrametric, reflecting the absence of a strict molecular clock in real data.

Sequence alignments for each simulated gene tree were generated using bppseqgen under a GTR+G4 substitution model, with model parameters estimated using bppml from a subset of avian genes containing all taxa.

1.4 48tax-sim

The avian simulation dataset was based on the published study (6). Model conditions were designed to reflect the four partitions of the biological dataset: exons-only, UCEs-only, introns-only, and long introns-only (loci $\geq$ 10,000 sites), each differing in average bootstrap support. Sequence alignments of lengths 250 bp, 500 bp, 1000 bp, and 1500 bp were simulated to approximate the bootstrap support values observed in the corresponding partitions (27%, 37%, 51%, and 60%, closely matching the empirical values of 24%, 39%, 48%, and 59%).

Shorter alignments were generated by trimming the longest (1500 bp) alignments, retaining the first 250 bp, 500 bp, or 1000 bp. This approach ensured that variations in gene tree support arose solely from alignment length differences. Compared to the mammalian simulation, the avian simulation represents a more challenging scenario due to higher gene tree estimation error and greater incomplete lineage sorting (ILS).

To evaluate the impact of dataset size, 20,000 gene trees were simulated, from which subsets were sampled without replacement to construct datasets of 200 genes (20 replicates), 500 genes (20 replicates), 1000 genes (20 replicates), and 2000 genes (10 replicates). Larger datasets were explored because the model conditions are challenging, and even with 2000 genes, reconstructed species trees may still exhibit considerable error.

Finally, a large-scale simulation of 14,350 genes was generated to match the characteristics of the real avian dataset in terms of the number of loci and distribution of bootstrap support: 8250 exon-like genes, 2500 intron-like genes, and 3600 UCE-like genes.

1.5 200tax-sim

The simulated 200 taxon dataset was based on the published study (7). This dataset was simulated using SimPhy to generate species and gene trees, and INDELible to simulate nucleotide sequence evolution along the gene trees. The resulting gene alignments were used to estimate gene trees, which were subsequently analyzed with coalescent-based species tree inference methods.

Species trees were simulated under a Yule speciation process using SimPhy, with the number of taxa fixed at 200. Tree characteristics were controlled by two parameters: maximum tree length and speciation rate, which together determine overall tree shape and the level of incomplete lineage sorting (ILS). Tree lengths of 500,000, 2,000,000, and 10,000,000 generations were considered, combined with speciation rates of $1e^{-6}$ and $1e^{-7}$ per generation. Shorter tree lengths produced shorter internal branches, leading to higher ILS, whereas longer trees reduced ILS. Higher speciation rates generated events closer to the tips, while lower rates shifted events toward the root. These parameter combinations created species trees with varying evolutionary difficulty, resulting in gene tree discordance ranging from approximately 10% to 70%, measured using the Robinson–Foulds (RF) distance between true gene trees and the species tree.

For each model condition, 50 independent species tree replicates were simulated, and for each species tree, 1000 gene trees were generated under the multispecies coalescent (MSC) model, assuming a constant effective population size of 200,000. SimPhy also introduced rate heterogeneity across genes and deviations from a strict molecular clock when converting gene tree branch lengths to mutation units.

Sequence evolution was simulated using INDELible, generating indel-free nucleotide alignments under a GTR+γ substitution model. Sequence lengths were determined by sampling μ uniformly from (5.7, 7.3) and σ from (0, 0.3), and then drawing each gene's length from a log-normal distribution parameterized by these values, resulting in average sequence lengths ranging approximately from 300 bp to 1500 bp. Substitution model parameters for GTR+γ were sampled from Dirichlet distributions with hyperparameters estimated via maximum likelihood from empirical datasets.

Gene trees were estimated from the simulated alignments using FastTree-II, which has comparable accuracy to more computationally intensive ML programs such as RAxML but is substantially faster. The resulting gene trees exhibited varying levels of gene tree estimation error, influenced primarily by ILS levels and species tree shape, with higher ILS and deeper speciation events producing greater error. Polytomies arising from insufficient sequence resolution were filtered by removing any gene tree with more than 50% unresolved internal nodes; replicates with insufficient remaining gene trees were excluded from downstream analyses.

In our study, we used the 200-taxon dataset with a tree length of 2M and a speciation rate of $1e^{-6}$. We analyzed 10 replicates from the 50 available.

1.6 500tax-sim

The simulated 500-taxon dataset was also obtained from the study of (7). The simulation procedure was identical to that used for the 200tax-sim dataset described above, including the use of SimPhy for species and gene tree simulation and INDELible for sequence evolution. The only difference was the number of taxa in the species trees, which was fixed at 500 instead of 200. All other parameters, including tree lengths, speciation rates, population size, number of replicates, and the number of gene trees per replicate, remained the same. For the 500-taxon dataset, we used the same settings as the 200-taxon dataset, with a tree length of 2M and a speciation rate of $1e^{-6}$. We analyzed 10 replicates from the 50 available.

2 Supplementary Tables

Table A: Average triplet score (TS) of the estimated species trees with respect to the corresponding sets of estimated gene trees across the datasets analyzed in this study

**Dataset**	**Model Cond^n**	**STELAR**						
		**MAD**	**MP**	**MV**	**OG**	**RAND**	**RD**	**RD-EX**
**11-taxon**	strong-5		641.6	641.6	761.65	533.947		
	strong-15		1930.45	1930.45	2291.1	1851.1		
	strong-25		3198.6	3198.6	3798.6	3149.2		
	strong-50		6389.6	6389.6	7589.8	6352.3		
	strong-100		12764.4	12764.4	15165.05	12733.9		
**15-taxon**	100-100	17235.2	17695.4	17695.4	17695.4	16457.4	17675.7	16868.3
	100-1000	20110.1	20290.5	20290.5	20290.5	18536.9	20340.2	19806.4
	100-true		23959.9	22207.4	32822.3	19874.4		
	1000-100	171608.1	175925.6	175925.6	175925.6	163764.7	175895.2	167931.7
	1000-1000	198719.1	199613.3	199613.3	199613.3	182300.7	201799.8	194817.4
	1000-true		241057.2	218618.5	327645.8	203841.5		
**37-taxon**	0.5X-200-500	989474.5	989467.45	989148.2	989344.1	874149.65	843120.75	981842.95
	1X-200-1000	1158433.25	1158536.85	1158536.85	1158536.3	961302.25	940074.8	1138218.45
	1X-200-500	1101439.55	1101722.65	1101557.0	1102434.0	952731.8	932451.85	1094015.15
	1X-200-true	1410709.85	1410709.85	1410709.85	1410709.85	998847.45		
	1X-400-1000	2314597.85	2314598.0	2314598.0	2314598.0	1926274.9	1925994.8	2275069.85
	1X-400-500	2202684.211	2202711.0	2202711.0	2202709.421	1905627.263	1905137.263	2184632.263
	1X-400-true	2820623.75	2820623.75	2820623.75	2820623.75	1998009.45		
	1X-800-1000	4624759.05	4624759.05	4624759.05	4624759.05	3855959.8	3855226.2	4546550.85
	1X-800-500	4415965.4	4415965.4	4415965.4	4415965.4	3810705.75	3809699.85	4376343.25
	1X-800-true	5479549.1	5643918.0	5643918.0	5643918.0	3997968.1		
	2X-200-500	1152943.65	1154764.9	1153979.25	1154896.6	988994.0	967021.4	1145624.8
**48-taxon**	0.5X-1000-500	9500062.25	10357359.35	9574800.2	10608691.5	7930725.65	7518799.15	10449514.2
	1X-100-500	720083.6	1068425.35	884224.4	1177023.25	852615.85	833157.05	1048816.6
	1X-1000-500	7606195.8	11379455.1	8782297.3	11723809.35	8499165.85	8171234.05	11123677.3
	1X-200-500	1500354.9	2213331.85	1745214.8	2352178.85	1712047.25	1649962.45	2203511.45
	1X-25-500	196975.8	270455.75	219774.65	298140.6	215021.0	212741.2	253673.0
	1X-50-500	365375.65	543951.15	453327.45	591600.8	427917.35	420172.9	517154.55
	1X-500-500	3797144.3	5693187.55	4361695.9	5862315.2	4311531.4	4099042.55	5528409.1
	2X-1000-500	6965546.05	11458453.75	7945845.2	11915250.75	8947757.9	8574869.6	10978527.2
**200-taxon**	estimated	755349429.111	765279555.556	749798311.556	759675755.889	785804351.333		
**Amniota**	Nucleotide	14818	15357	14922	14992	17869	15019	15476
**Angiosperm**	Nuclear	1214602	1200657	1200657	1200657	867932		

Table B: Average triplet score (TS) of the estimated species trees with respect to the corresponding sets of true gene trees across the datasets analyzed in this study

**Dataset**	**Model Cond^n**	**STELAR**						
		MAD	MP	MV	OG	RAND	RD	RD-EX
**11-taxon**	strong-5		660.35	660.35	780.4	558.4		
	strong-15		2044.9	2044.9	2407.3	1936.35		
	strong-25		3425.05	3425.05	4025.05	3329.9		
	strong-50		6859.25	6859.25	8050.7	6748.0		
	strong-100		13726.8	13726.8	16116.95	13601.95		
**15-taxon**	100gt-100bp	30030.818	30461.9	30461.9	30461.9	20156.0	28224.7	21472.9
	100gt-1000bp	32766.9	32521.4	32521.4	32521.4	20864.3	26324.6	23831.8
	1000gt-100bp	321010.5	309185.1	309185.1	309185.1	198069.8	289730.0	217874.7
	1000gt-1000bp	327645.8	326824.3	326824.3	326824.3	199191.7	262838.1	231898.8
**37-taxon**	0.5X-200-500	1248086.15	1248052.1	1248244.05	1248109.45	920812.15	920358.95	1149700.0
	1X-200-1000	1410385.05	1410652.65	1410652.65	1410650.6	998723.7	832956.55	1281463.75
	1X-200-500	1410381.45	1410467.05	1410405.45	1409539.75	998074.45	869667.85	1291754.75
	1X-400-1000	2820598.0	2820594.95	2820594.95	2820594.95	1997631.55	1997081.55	2563032.95
	1X-400-500	2820118.789	2820230.579	2820230.579	2820224.316	1996615.684	1995856.0	2562692.579
	1X-800-1000	5479549.1	5479549.1	5479549.1	5479549.1	3881310.75	3147458.75	4978768.85
	1X-800-500	5479505.2	5479505.2	5479505.2	5479505.2	3881010.3	3371166.75	4978624.65
	2X-200-500	1497359.75	1497454.1	1498962.2	1496526.2	1034799.55	1034311.6	1357399.2
48-taxon	0.5X-1000-500	10051174.3	11095538.9	10139002.25	11530857.55	8288721.45	7824656.45	11219496.35
	1X-100-500	732817.85	1127397.95	918274.75	1251100.15	897977.55	877085.75	1106691.05
	1X-1000-500	7840046.25	12066355.55	9109073.35	12591978.75	8972138.9	8617815.35	11802114.85
	1X-200-500	1538912.75	2338739.1	1812038.7	2506970.35	1803167.7	1736660.8	2326202.4
	1X-50-500	372201.95	571365.9	469727.05	624282.25	449897.6	441395.05	543559.8
	1X-500-500	3909483.9	6035359.7	4527019.1	6280826.55	4547023.75	4318498.2	5869280.75
	2X-1000-500	7213265.45	12494254.9	8357037.45	13361902.55	9542505.2	9084815.15	11773397.15

Table C: Average quartet score (QS) of the estimated species trees with respect to the corresponding sets of estimated gene trees across the datasets analyzed in this study.

Dataset	Model Cond^n	ASTRAL	STELAR						
			MAD	MP	MV	OG	RAND	RD	RD-EX
**11-taxon**	strong-100	26705.4		26705.4	26705.4	26702.7	26535.85		
	strong-15	4068.25		4066.6	4066.6	4064.6	3970.7		
	strong-25	6695.4		6695.15	6695.15	6695.15	6596.05		
	strong-5	1357.4		1356.9	1356.9	1355.35	1318.9		
	strong-50	13383.1		13383.05	13383.05	13380.1	13190.25		
**15-taxon**	100gt-100bp	69933.8	69686.3	69276.5	69276.5	69276.5	68780.0	69545.4	68252.7
	100gt-1000bp	82166.6	82105.2	82013.2	82013.2	82013.2	80610.5	81830.5	81233.9
	100gt-true	84634.9		84516.7	84570.0	84634.9	83301.7		
	1000gt-100bp	693656.1	691299.8	684572.4	684572.4	684572.4	682891.75	685192.0	687215.4
	1000gt-1000bp	818022.2	817937.3	817532.111	817532.111	817532.111	811553.333	815358.222	811824.1
	1000gt-true	844184.3		844184.3	844184.3	844184.3	833455.6		
**37-taxon**	0.5X-200-500	10007425.55	10007255.25	10007260.7	10007291.35	10007287.65	9999226.7	9995322.45	10006075.75
	1X-200-1000	11586641.45	11586613.85	11586550.25	11586550.25	11586540.25	11581925.35	11578626.2	11586006.4
	1X-200-500	11270657.55	11270311.8	11270455.75	11270303.45	11270534.45	11262629.65	11257710.1	11269601.25
	1X-200-true	11746203.5	11746194.95	11746198.85	11746198.85	11746198.85	11741292.0		
	1X-400-1000	23159900.85	23159890.65	23159900.85	23159900.85	23159900.85	23148064.55	23144744.4	23159090.3
	1X-400-500	22530090.421	22529758.158	22530050.053	22530050.053	22529985.526	22518243.421	22502535.105	22528736.053
	1X-400-true	23487558.65	23487558.65	23487558.65	23487558.65	23487558.65	23481670.65		
	1X-800-1000	46342464.15	46342464.15	46342464.15	46342464.15	46342464.15	46329619.95	46307948.85	46342464.15
	1X-800-500	45096874.45	45096874.45	45096874.45	45096874.45	45096874.45	45083925.95	45052620.65	45096285.3
	1X-800-true	45632640.45	45632640.45	45632640.45	45632640.45	45632640.45	45621015.4		
	2X-200-500	11947266.5	11945264.3	11947184.75	11947089.25	11947252.75	11943092.85	11939246.25	11947046.35
**48-taxon**	0.5X-1000-500	110583148.55	103668933.6	109362112.0	104025102.85	110383433.15	109578470.35	109734374.2	110314684.7
	1X-100-500	12383876.5	12025621.5	12138032.25	11964202.2	12318052.45	12207533.7	12179035.15	12204053.45
	1X-1000-500	123024287.2	119274515.2	121790825.05	114929114.55	122916649.8	121998800.7	122241813.9	122035658.75
	1X-200-500	24713063.7	23962846.1	24378714.6	23863357.3	24642898.2	24418292.65	24372977.5	24465457.3
	1X-25-500	3150240.45	3052890.9	3051834.85	3043066.7	3115783.05	3067542.55	3054728.5	3093191.25
	1X-50-500	6234610.35	6065409.95	6058419.0	6010449.1	6182000.55	6122005.95	6084794.85	6118501.55
	1X-500-500	61574240.65	59710450.05	60903919.2	58540593.6	61477542.2	60979019.25	61104007.6	61057573.4
	2X-1000-500	131631393.9	129156563.95	129905861.95	126831800.25	131546355.5	130155966.9	130740197.05	130252231.35
**200-taxon**	estimated	47622712046.3	47536451073.2	47446554046.6	47519602308.4	47615850756.2	47390448761.7		
**Amniota**	Nucleotide	97890	97735	97735	97881	97890	97890	93067	94165
**Angiosperm**	Nuclear	11553053	10963619	10936281	10936281	10936281	11210579		

Table D: Average quartet score (QS) of the estimated species trees with respect to the corresponding sets of true gene trees across the datasets analyzed in this study

Dataset	Model Cond^n	ASTRAL	STELAR						
			MAD	MP	MV	OG	RAND	RD	RD-EX
**11-taxon**	strong-100	30708.3		30708.3	30708.3	30663.25	30039.25		
	strong-15	4513.6		4517.15	4517.15	4533.65	4273.95		
	strong-25	7622.3		7625.5	7625.5	7625.5	7283.15		
	strong-5	1391.85		1386.95	1386.95	1387.5	1371.4		
	strong-50	15322.95		15327.75	15327.75	15282.75	14743.55		
**15-taxon**	100gt-100bp	82158.6	83019.8	80525.5	80525.5	80525.5	78400.5	80608.9	78943.1
	100gt-1000bp	84345.1	84545.7	84127.9	84127.9	84127.9	82597.5	83759.7	83369.4
	1000gt-100bp	827970.1	8421420	816941.9	816941.9	816941.9	815221.4	808329.0	823085.8
	1000gt-1000bp	843362.8	844184.3	843362.8	843362.8	843362.8	837052.9	839741.3	837294.5
**37-taxon**	0.5X-200-500	10311630.05	10312053.55	10311354.45	10311638.25	10311920.2	10304742.0	10296194.95	10311070.2
	1X-200-1000	11743809.5	11743714.8	11744886.3	11744886.3	11744820.7	11739393.55	11739601.85	11743395.4
	1X-200-500	11739721.0	11740608.15	11740822.35	11740575.95	11740228.2	11734417.9	11729777.7	11739398.1
	1X-400-1000	23486728.75	23486827.8	23486728.75	23486728.75	23486728.75	23470981.1	23467004.0	23486217.7
	1X-400-500	23482457.0	23479373.16	23481661.05	23481661.05	23481461.05	23468128.37	23445782.8	23478993.4
	1X-800-1000	45632640.45	45632640.45	45632640.45	45632640.45	45632640.45	45619021.2	45608973.15	45632640.4
	1X-800-500	45631302.8	45631302.8	45631302.8	45631302.8	45631302.8	45610570.45	45596005.65	45629582.8
	2X-200-500	12565281.6	12560555.4	12567292.7	12568800.8	12565175.0	12559268.9	12547261.1	12568200.0
**48-taxon**	0.5X-1000-500	119513731.6	110530209.8	117731361.0	110982521.8	119135686.95	118129439.45	118354949.2	119030503.3
	1X-100-500	13567423.65	13051253.55	13248847.0	12954507.65	13454157.6	13359236.1	13315353.65	13340893.3
	1X-1000-500	135985986.4	129816269.5	133918071.6	124794923.4	135761706.2	134327102.0	134657697.5	134271816.7
	1X-200-500	27170167.45	25974395.8	26665882.2	25855260.35	27003290.0	26767338.2	26695842.15	26745445.5
	1X-50-500	6776224.95	6549690.9	6565844.35	6482442.4	6708723.9	6668536.7	6611222.05	6653193.35
	1X-500-500	67891776.05	64911557.35	66810392.75	63519122.5	67684969.8	66998284.4	67178572.9	67077301.2
	2X-1000-500	148393885.35	143157302.05	144655982.55	139243603.85	148183540.35	145197013.0	146399056.5	145292248.1

3 Results for SuperTriplets

To assess whether the observed sensitivity patterns to gene tree rooting extend beyond STELAR, we conducted parallel experiments using the triplet-based species tree estimation method *SuperTriplets* (8).

3.1 37tax-sim

We applied the same experimental settings as described for STELAR on the 37tax-sim dataset. Aside from the RD-EX method performing relatively worse under SuperTriplets (frequently yielding the second-worst result), the average Robinson-Foulds distances exhibit trends consistent with those observed for STELAR, supporting the generalizability of our conclusions to other triplet-based methods.

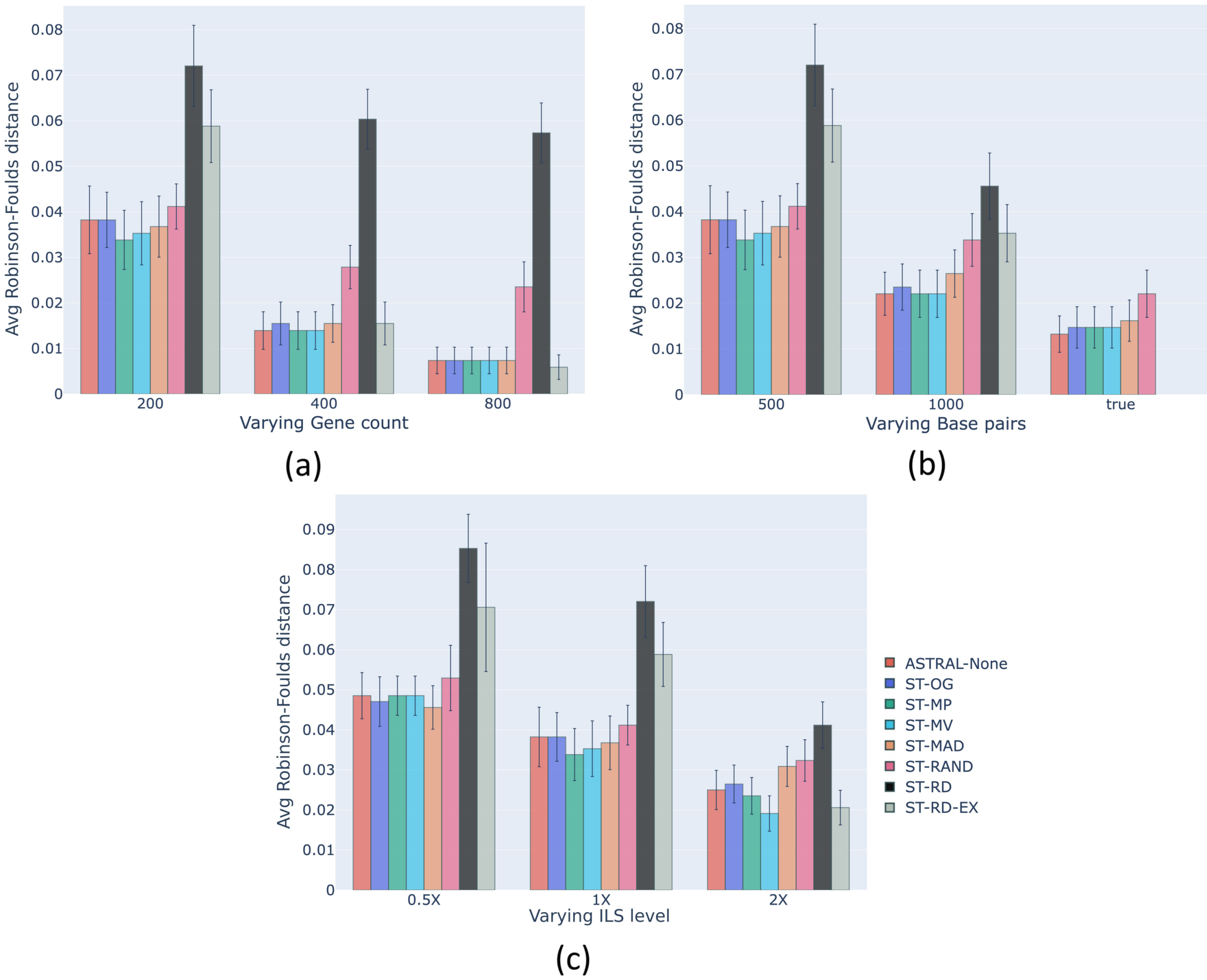

Fig A: Comparison of average Robinson–Foulds distance (± standard error over 20 replicates) for *SuperTriplets* under different gene tree rooting methods on the 37tax-sim mammalian dataset. (a) Gene count varied (200, 400, 800) while fixing ILS at moderate (1X) and sequence length at 500bp. (b) Sequence length varied (500bp, 1000bp, true-gt) while fixing ILS at moderate (1X) and gene count at 200. (c) ILS level varied (0.5X, 1X, 2X) while fixing gene count at 200 and sequence length at 500bp.

3.2 15tax-sim

We ran experiments with SuperTriplets under the same conditions as STELAR for the 15tax-sim dataset. The RootDigger variants (RD and RD-EX) showed improved performance in difficult conditions (100bp) when evaluated with SuperTriplets. In contrast to the STELAR results, they outperformed OG, MP, and MV in these cases. Furthermore, unlike STELAR, the trees inferred by SuperTriplets when paired with MP, MV, and OG were not identical in these conditions, resulting in slightly different RF scores. Other than these deviations, the fundamental trends remained the same, with MAD outperforming all other variants in all model conditions. This reaffirms that the rooting impacts were not STELAR-specific.

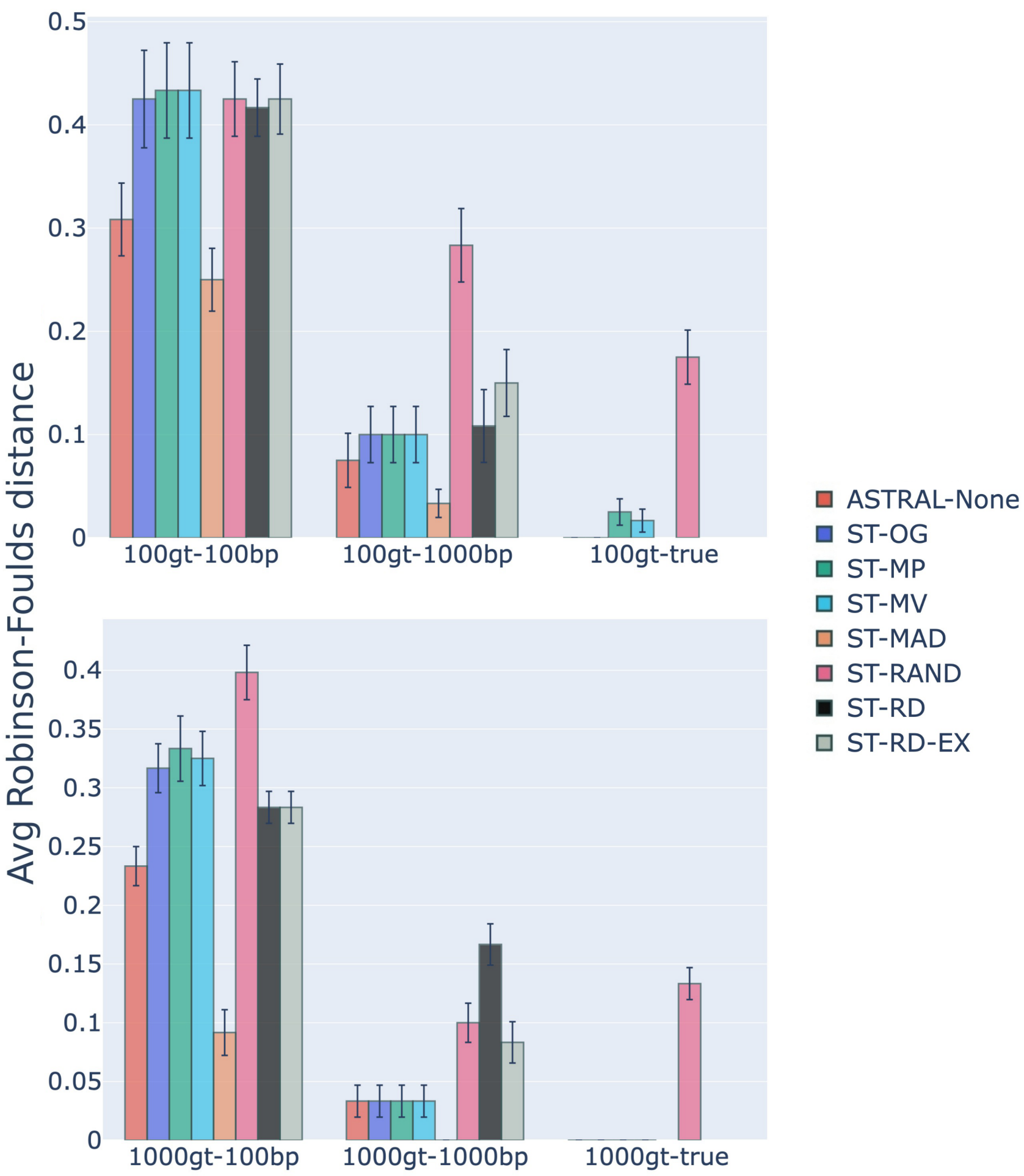

Fig B: Comparison of average Robinson-Foulds distance (with standard error bars) for *SuperTriplets* under different rooting methods on the 15tax-sim dataset. We varied the number of estimated gene trees (100 to 1000 genes) as well as the sequence length (100bp to 1000bp). Model conditions with true gene trees were also analyzed; however, due to the absence of branch lengths, we could not run MAD on these cases (100-true and 1000-true)

3.3 11tax-sim

We report the RF scores (Fig C) of SuperTriplets, paired with different rooting methods on the 11tax-sim dataset. Minor variation is noticable among the variants in the more "difficult" conditions of 5, 15, and 25 genes, but all other variants outperform OG rooting in the easier 50, 100 gene count model conditions, similar to STELAR. ST-RAND performs the worst in all cases, but the other Supertriplets variants stay competitive with ASTRAL.

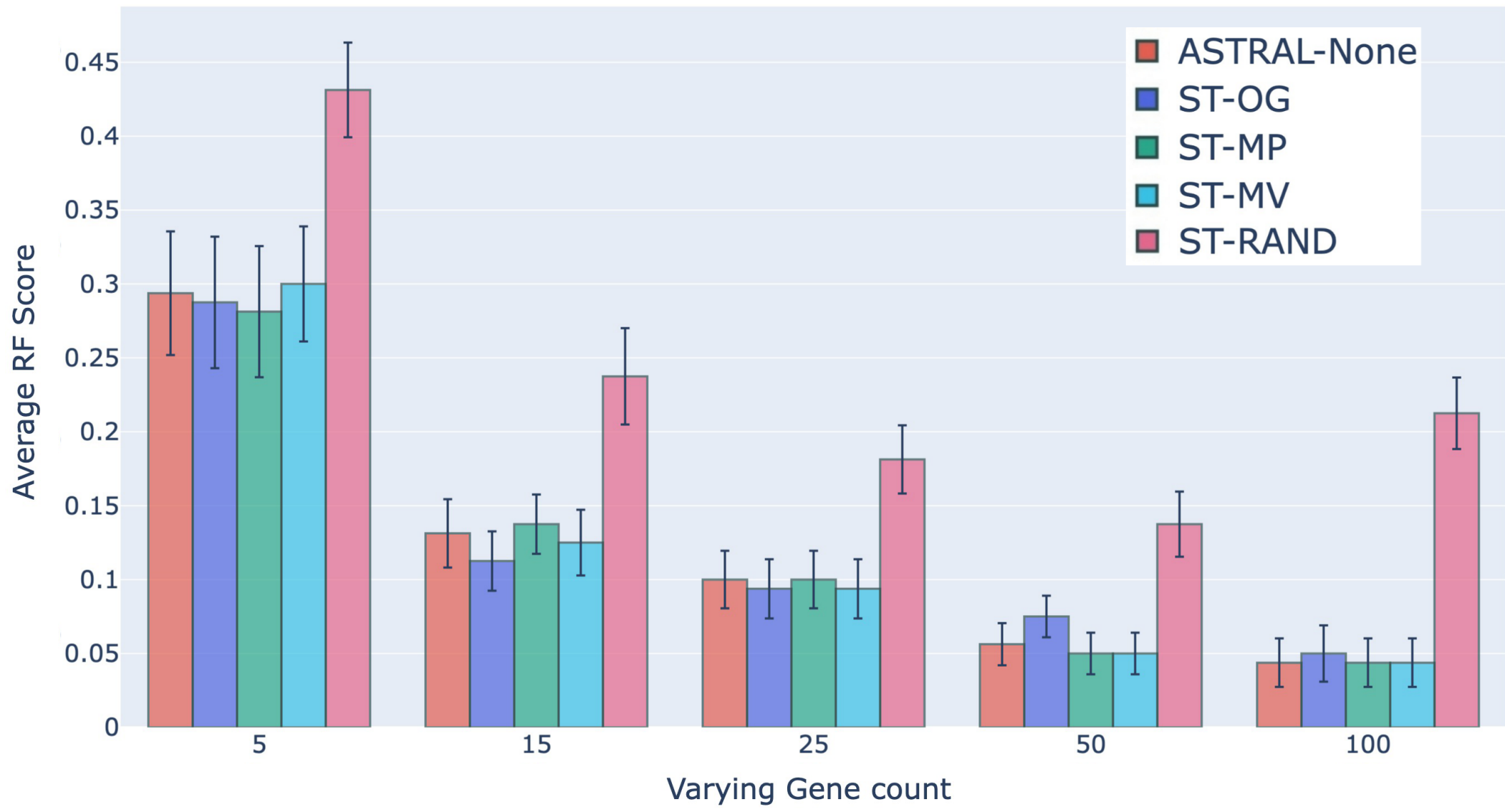

Fig C: Comparison of the Robinson-Fould's distance for ASTRAL and the SuperTriplets variants on the 11tax-sim dataset. Gene counts were varied from 5 to 100 for high ILS.

3.4 48tax-sim

We also share results of SuperTriplets on the 48tax-sim dataset. Similar to STELAR, random rooting performs surprisingly well on this dataset, with the worst performances from the MV and MAD variants. On a different note, a noticable improvement in performance can be observed in case of the RootDigger variants. As such, ST-RD and ST-RD-EX can be seen putting up competitive scores in the more difficult model conditions.

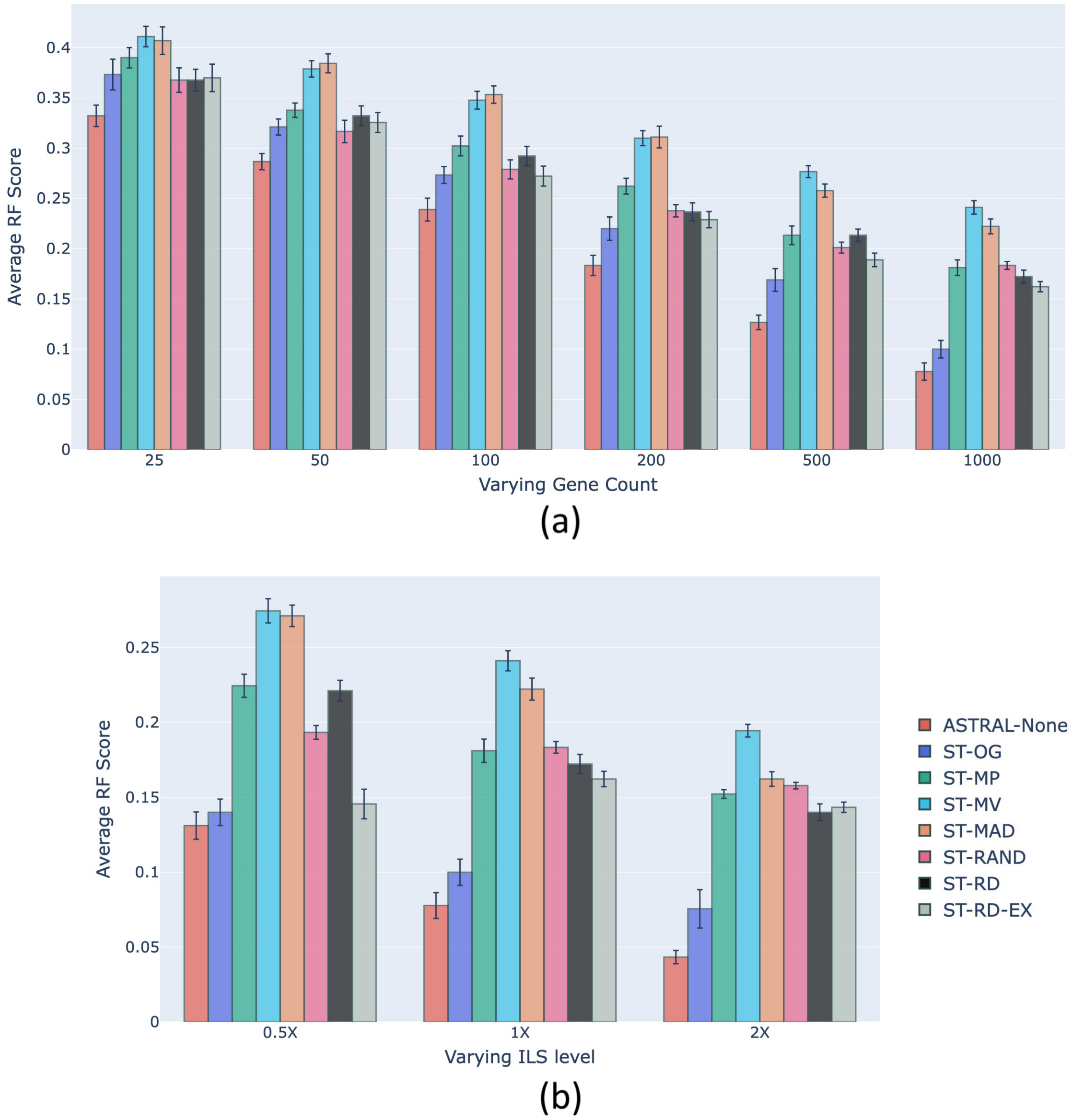

Fig D: Comparison of Average Robinson-Foulds distance for ASTRAL and SuperTriplets with different rooting methods for the 48tax-sim avian dataset. (a) We fixed the ILS at moderate (1X) and the base pairs at 500bp, and varied gene counts between from 25 to 1000. (b) We set the gene trees at 1000, the sequence length at 500bp, and varied the ILS between high (0.5X), moderate (1X) and low (2X)

3.5 Larger Datasets (200tax-sim and 500tax-sim)

We repeated the comparison between ASTRAL and SuperTriplet variants on the 200 and 500 taxa datasets as well (Fig E). The RF scores on the 200tax-sim dataset (Fig E(a)) were almost identical to the ones obtained from STELAR on the same conditions.

The performance of SuperTriplets variants was consistently better than their STELAR counterparts in the 500tax-sim dataset (Fig E(b)). In the model condition with estimated gene trees, ST-RAND gave the best scores among the ST variants, almost catching up to ASTRAL in terms of RF-scores. However, in one replicate in the true gene trees model condition, ST-RAND yielded poor a RF score, thus skewing its performance in this case.

It is worth noting that, for the model condition with true gene trees for the 500tax-sim dataset, ST-MP yielded an unexpectedly high RF score which was skewing its performance. As such, we analysed results on this model condition over the other 9 replicates (except R4).

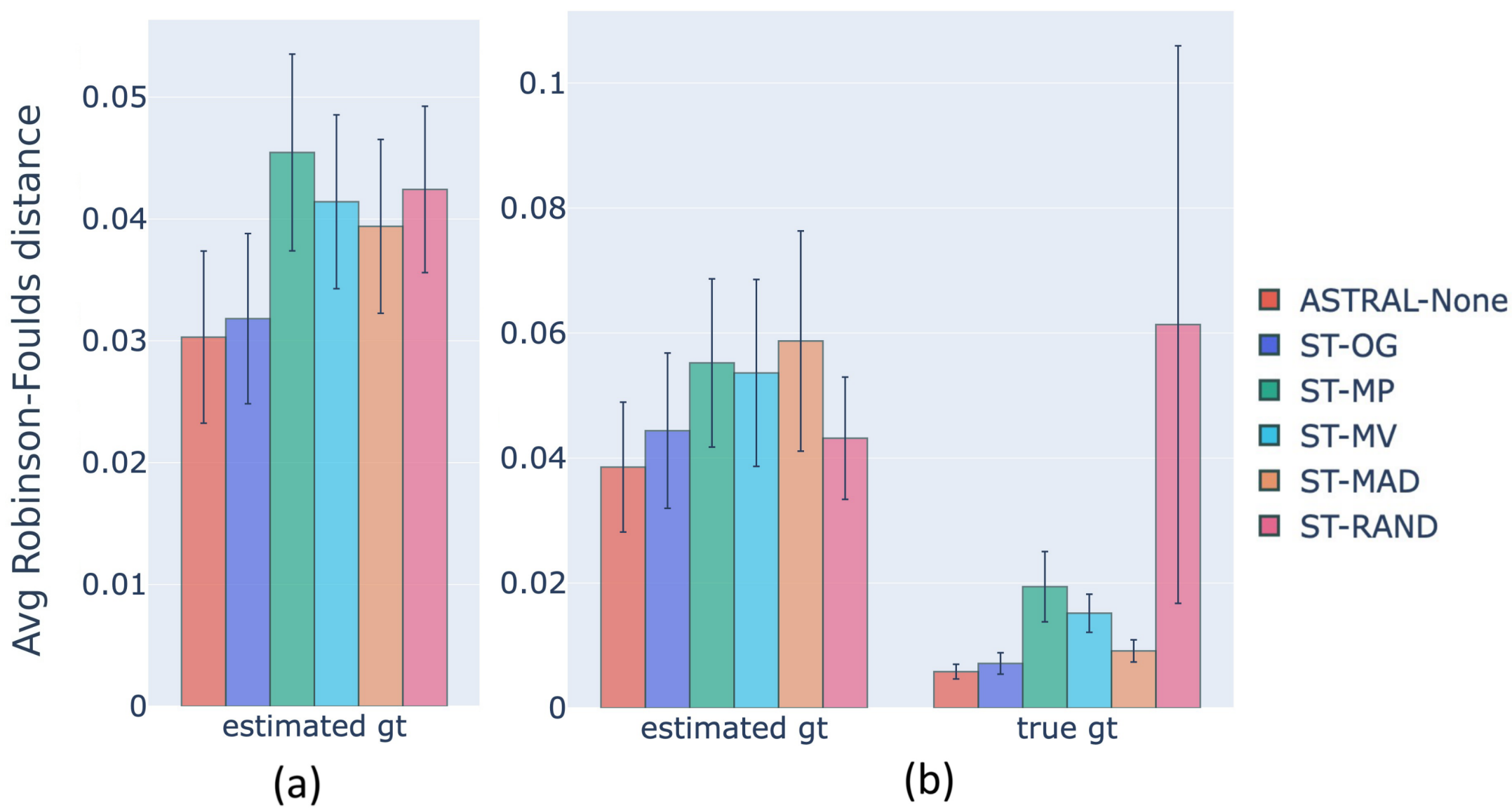

Fig E: Comparison of average Robinson-Foulds distance for ASTRAL and SuperTriplets with different rooting methods for higher-order taxa datasets. (a) Comparison of the RF distance for 200tax-sim dataset (b) Comparison of the RF distance for 500tax-sim dataset, with estimated gene trees and true gene trees respectively. For the true gt model condition, we analysed 9 replicates (except R4), since an unexpectedly high RF score from ST-MP was skewing its overall results in this case.

4 Results on the Biological Mammalian Dataset

Supplementary Fig F shows the inferred mammalian species tree topology, rooted at Gallus. As discussed in the main text (Section 4.2.3), all methods recovered an identical unrooted topology, differing only in the placement of the root. The tree is therefore shown under the biologically supported Gallus rooting for clarity and reference.

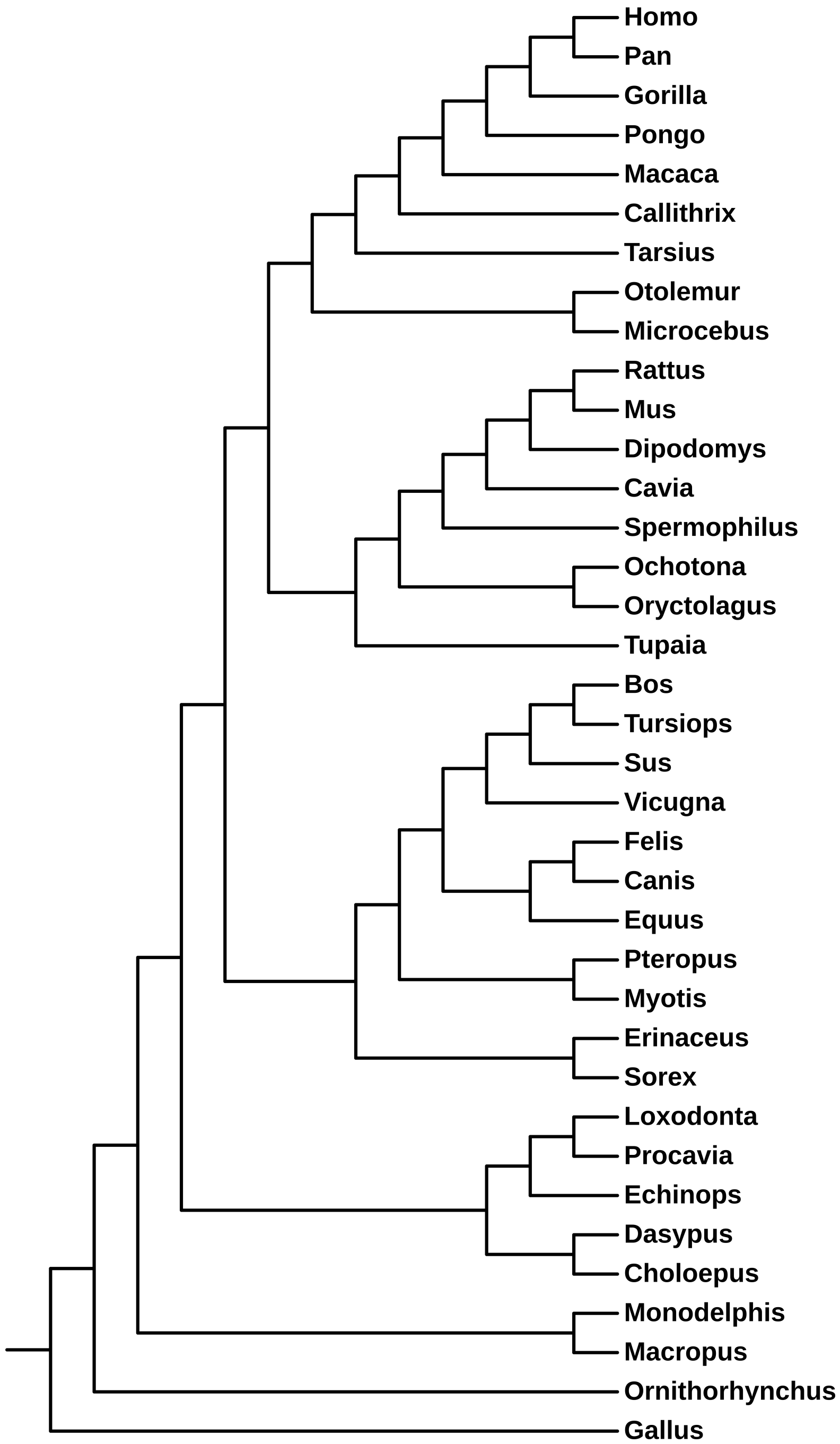

Fig F: Estimated species tree for Mammalian biological dataset by ASTRAL and the MV, MV, OG, MAD, RD, RD-EX, and RAND variants of STELAR. All the inferred trees have been rerooted at Gallus for ease of comparison.

References

[1] Chung Y, Ané C. Comparing two Bayesian methods for gene tree/species tree reconstruction: A simulation with incomplete lineage sorting and horizontal gene transfer. Syst Biol. 2011;60(3):261-75.

[2] Price MN, Dehal PS, Arkin AP. FastTree 2 – Approximately maximum-likelihood trees for large alignments. PLoS ONE. 2010;5(3):e9490. doi:10.1371/journal.pone.0009490.

[3] Stamatakis A. RAxML-NI-HPC: Maximum likelihood-based phylogenetic analyses with thousands of taxa and mixed models. Bioinformatics. 2006;22:2688-90.

[4] Heled J, Drummond AJ. Bayesian inference of species trees from multilocus data. Mol Biol Evol. 2010;27:570-80.

[5] Bayzid MS, Mirarab S, Boussau B, Warnow T. Weighted statistical binning: enabling statistically consistent genome-scale phylogenetic analyses. PLoS ONE. 2015;10(6).

[6] Mirarab S, Bayzid MS, Boussau B, Warnow T. Statistical binning enables an accurate coalescent-based estimation of the avian tree. Science. 2014;346(6215):1250463.

[7] Mirarab S, Warnow T. ASTRAL-II: coalescent-based species tree estimation with many hundreds of taxa and thousands of genes. Bioinformatics. 2015;31(12):i44-52.

[8] Ranwez V, Criscuolo A, Douzery EJ. SuperTriplets: a triplet-based supertree approach to phylogenomics. Bioinformatics. 2010;26(12):i115-23.
